# A trispecific antibody targeting EGFR/cMET/VEGF-A demonstrates multiple mechanisms of action to inhibit wild-type and mutant NSCLC animal models

**DOI:** 10.3389/fonc.2025.1533059

**Published:** 2025-05-16

**Authors:** Ying Jin, Ping Sun, Peng Chen, Yuqiang Xu, Guangmao Mu, Zhengxia Zha, Simin Wu, Meixia Fu, Hao Jiang, Sheng Huang, Fulai Zhou, Chao Han, Mark L. Chiu

**Affiliations:** ^1^ Research & Development Department, Tavotek Biotherapeutics, Suzhou, Jiangsu, China; ^2^ Research & Development, Tavotek Biotherapeutics, Spring House, PA, United States

**Keywords:** trispecific antibodies, NSCLC, EGFR cancer cells +, cmet, VEGF

## Abstract

**Introduction:**

Non-small cell lung cancer (NSCLC) patients who do not respond to standard of care treatment can have activating mutations in the epidermal growth factor receptor (EGFR) and mesenchymal epithelial transition factor (cMET) signaling pathways, as well as having enhanced levels of vascular endothelial growth factor (VEGF). To combat such resistance mechanisms, TAVO412, was engineered to control aberrant cMET, VEGF-A, and EGFR activities.

**Methods:**

*In vitro* assays assessed TAVO412’s cell binding, ligand blockade, phosphorylation inhibition, and Fc effector functions. In vivo efficacy was evaluated in NSCLC xenograft models, with subsequent tumor resection for ex vivo quantification of EGFR and cMET levels.

**Results:**

TAVO412 robustly suppressed ligand-induced phosphorylation of EGFR and cMET in NSCLC cell lines. TAVO412 demonstrated more potent antitumor activity than amivantamab and cetuximab in NSCLC xenograft models using cell lines with varying levels of mutant and wild-type *EGFR* and *cMET*. In addition, TAVO412 had both EGFR/ cMET receptor degradation and enhanced Fc effector functions for tumor cell cytotoxicity. Moreover, TAVO412 in combination with osimertinib, lazertinib, docetaxel, and radiotherapy, resulted in complete and durable regression of NSCLC xenograft tumors.

**Discussion:**

These findings highlight TAVO412 as a promising therapeutic agent with multiple mechanisms of action and strong potential for synergistic combinations in NSCLC treatment.

## Introduction

Non-small cell lung cancer (NSCLC) is the most common type of lung cancer with a poor prognosis and low 5-year overall survival (OS) (1). Prognosis and treatment approaches are primarily influenced by histology, stage at diagnosis, and molecular abnormalities. Since standards of care therapies such as surgery, chemotherapy, and radiotherapy do not fully meet the medical needs, there are ongoing discovery of better targeted therapies to provide greater efficacy and safety especially for patients with specific genomic alterations (2). Epidermal growth factor receptor (*EGFR*) gene dysregulation accounts for 23 - 30% of the NSCLC activating mutations (3–5). About 90% of the dominant *EGFR* mutations are short, in-frame deletions of exon 19 (Ex19Del) and mutations at position 858 in exon 21 (L858R missense replacements); and 4-10% being *EGFR* exon 20 insertions (*EGFR* Ex20ins) (5, 6).

The first-line standards of care for *EGFR* mutation-positive NSCLC patients include tyrosine kinase inhibitors (TKIs) that have considerable higher efficacy than standard chemotherapy (7). However with the emerging NSCLC resistance arising from different mutations, subsequent generations of small molecule EGFR TKIs have been developed (8). Unfortunately, small-molecule kinase inhibitors are constrained because of their intrinsically limited molecular surface area for binding that can be rendered ineffective by receptor mutations; sometimes just a single amino acid change (9, 10). One approach to target a broader pool of mutant EGFR is to employ anti-EGFR antibodies that provide therapeutic benefits in NSCLC, but still fall short with ensuing development of resistance (5). The mesenchymal-epithelial transition factor (*cMET*) amplification has a role in the resistance mechanism in patients that are no longer responsive to EGFR-TKIs and anti-EGFR antibodies (11, 12). Thus, amivantamab, designed for dual inhibition of EGFR and cMET, was developed and demonstrated promising tumor inhibition activities in preclinical and clinical studies (13–15). Amivantamab, a fully human bispecific antibody (BsAb) that targets both EGFR and cMET, received FDA approval in May 2021 for the treatment of advanced or metastatic NSCLC with *EGFR* Ex20ins mutations (5, 16, 17). However, some patients treated with amivantamab experience limited progression-free survival (PFS) and eventual disease progression, while a subgroup of patients do not respond to the treatment (18). Thus, there remains a need for drugs that could help patients with innate and acquired resistance to these standard-of-care therapies.

TKI resistant NSCLC patients exhibit aberrant EGFR and cMET signaling as well as elevated vascular endothelial growth factor (VEGF) receptor pathway activity which is a critical driver of solid tumor angiogenesis (19). EGFR and the receptor for VEGF, VEGF receptor 2 (VEGFR-2), share common downstream pathways such that inhibition of one pathway can be compensated by the upregulation of the other. In addition, *EGFR*-mutant tumors are more dependent on VEGF-A signaling compared to *EGFR* wild-type tumors (20). VEGF-A has a dual role of promoting tumor cell proliferation through autocrine signaling and stimulating angiogenesis via paracrine mechanisms (21). In line with this mechanism, two anti-angiogenic agents were approved by the FDA for the treatment of advanced NSCLC: bevacizumab [an anti-VEGF-A monoclonal antibody (mAb)] and ramucirumab (an anti-VEGFR-2 mAb) (22, 23). To control EGFR and VEGF signal pathways, the dual-targeted approach combining erlotinib (EGFR TKI) with bevacizumab demonstrated superior antitumor activity compared to monotherapy in clinical practice, leading to its approval as a first-line treatment option for EGFR-mutant NSCLC (19, 24). In addition, dual inhibition of cMET and VEGFR-2 has shown strong inhibition of tumor growth and angiogenesis in xenograft models (25, 26). However, since these strategies still have a narrow therapeutic index, a more comprehensive treatment is required.

Considering the extensive crosstalk among the three pathways, the combined inhibition of the EGFR, cMET, and VEGF pathways could overcome resistance and could be an effective treatment approach for NSCLC patients. The aim of the current study was to demonstrate how TAVO412, a trispecific antibody targeting EGFR, cMET, and VEGF, controlled dysfunctional NSCLC tumor growth activities. TAVO412 was engineered to have differentiated mechanisms of action (MOA) that included ligand blocking, EGFR/cMET receptor phosphorylation inhibition, EGFR/c-MET receptor degradation, shutdown of angiogenesis, and enhanced Fc effector functions. These MOAs manifested in *in vivo* antitumor activities of TAVO412 in a diverse panel of NSCLC tumor models with varying *EGFR* mutations, a broad range of EGFR and cMET receptor densities, and VEGF secretion levels. In addition, TAVO412 was shown to have stronger anti-tumor activities in combination with other standards of care treatments that included radiotherapy, chemotherapy, and 3^rd^ generation EGFR TKIs.

## Results

### TAVO412 bound and inhibited ligand binding to NSCLC cell lines

**Figure 1:**
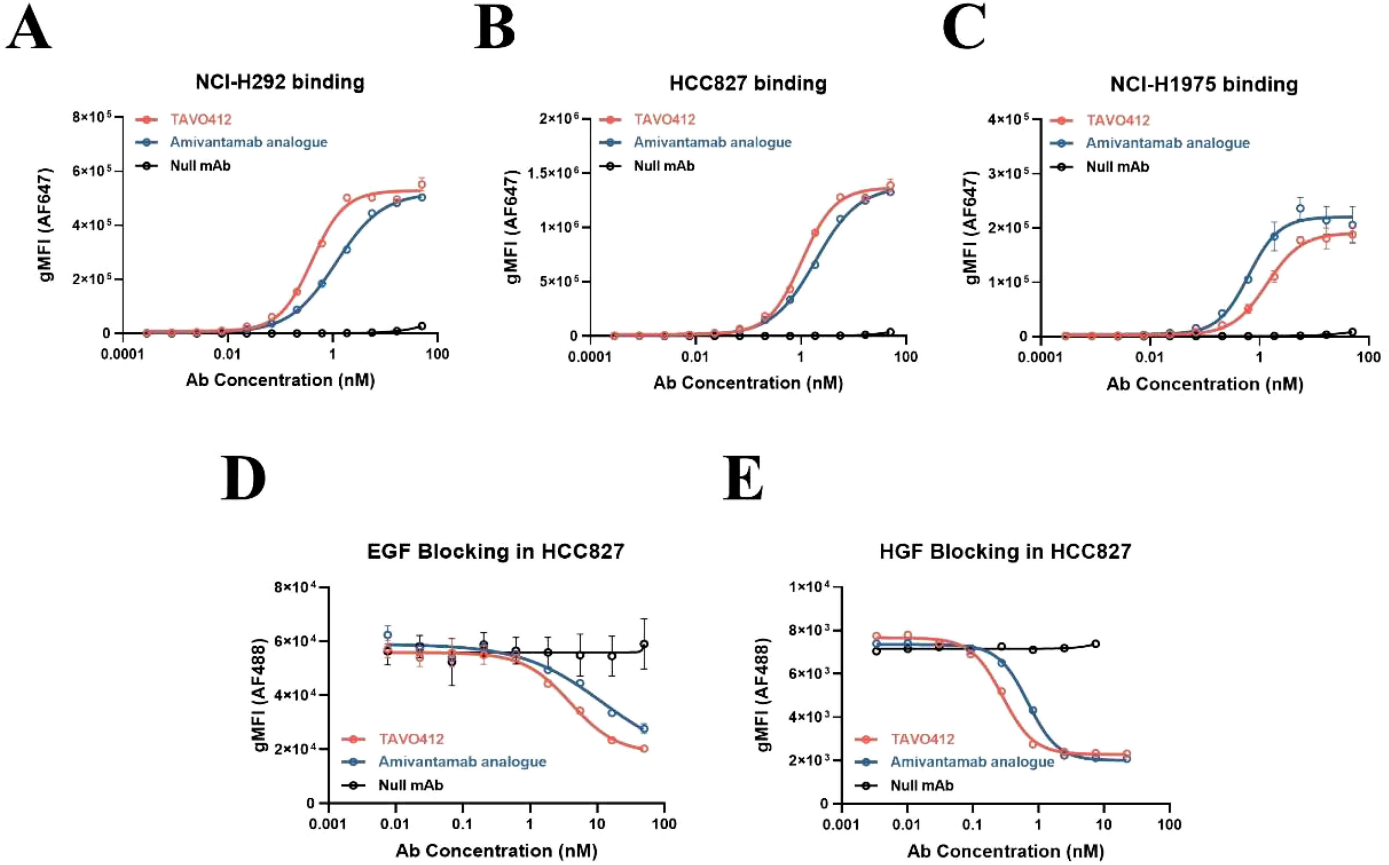
TAVO412 bound to NSCLC cell lines and blocked binding of EGF and HGF. The binding of TAVO412 (red open circle), Amivantamab analogue (blue open circle) and null mAb (black open circle) to **(A)** NCI-H292; **(B)** HCC827; and **(C)** NCI-H1975 NSCLC cell lines as analyzed by flow cytometry. See **Supplementary Table S2** for corresponding EC_50_, 95% CI for EC_50_, and efficacy (span in y axis). Blocking of **(D)** EGF and **(E)** HGF from binding to HCC827 cells was assessed by flow cytometry with TAVO412 (red open circle), Amivantamab analogue (blue open circle) and null mAb (black open circle). See **Supplementary Table S3** for corresponding IC_50_, 95% CI for IC_50_, and efficacy (span in Y-axis). The data from three independent experiments were expressed as the mean ± SEM of duplicate treatments. The amivantamab analogue served as a positive control molecule while the null mAb served as a negative control. The abbreviations were: gMFI, geometric mean fluorescent intensity; AF647, Alexa Fluor 647 dye; AF488, Alexa Fluor 488 dye; Ab, antibody; nM, nanomolar; EGF, epidermal growth factor; HGF, hepatocyte growth factor; SEM, standard error of the mean.

TAVO412 was produced in CHO cells and purified to high monomeric purity (>98%), as confirmed by SEC-HPLC and further validated by SDS-PAGE (**Supplementary Figures S1A, B**). The antibody demonstrated excellent thermal stability, with a melting temperature (Tm) of 65.2 ± 0.3°C (Tm1) and 74.6 ± 0.4°C (Tm2) determined by differential scanning fluorometry (DSF). TAVO412, the comparator amivantamab analogue, and a null control antibody binding to NSCLC cell lines were assessed by flow cytometry. The NSCLC cell lines (NCI-H292, HCC827, NCI-H1975, NCI-H460, NCI-H1299, NCI-H358, and NCI-H596) spanned different *EGFR* mutation profiles, a range of EGFR/cMET receptor densities, and varying levels of VEGF secretion (**Supplementary Table S1**). TAVO412 had high avidity binding with EC_50_ values that were similar to those of the amivantamab analogue (NCI-H292: 0.399 nM versus 1.150 nM; HCC827: 1.037 nM versus 1.885 nM; NCI-H1975: 1.358 nM versus 0.626 nM, for TAVO412 versus amivantamab analogue respectively) (**Figures 1A–C**; **Supplementary Figures S2A-D**; **Supplementary Table S2**). The levels of TAVO412 binding correlated with the EGFR receptor densities that were in higher levels than cMET receptor densities (data not shown).

The ability of TAVO412 to block EGF and HGF binding to their respective receptors was assessed using a cell-based flow cytometry assay in HCC827 cells(EGFR: cMET receptor density ratio = 14.5, **Supplementary Table S1**). TAVO412 effectively blocked EGF binding in comparison to the amivantamab analogue (IC_50_ of 4.013 nM versus 11.86 nM) and HGF binding (IC_50_ of 0.282 nM versus 0.689 nM) (**Figures 1D, E**; **Supplementary Table S3**). The non-binding (null) control antibody showed no inhibitory effects. The potency of TAVO412 in blocking VEGF-A binding to VEGFR2 was tested and has been reported elsewhere (manuscript submitted).

### TAVO412 inhibited ligand-induced receptor phosphorylation

**Figure 2:**
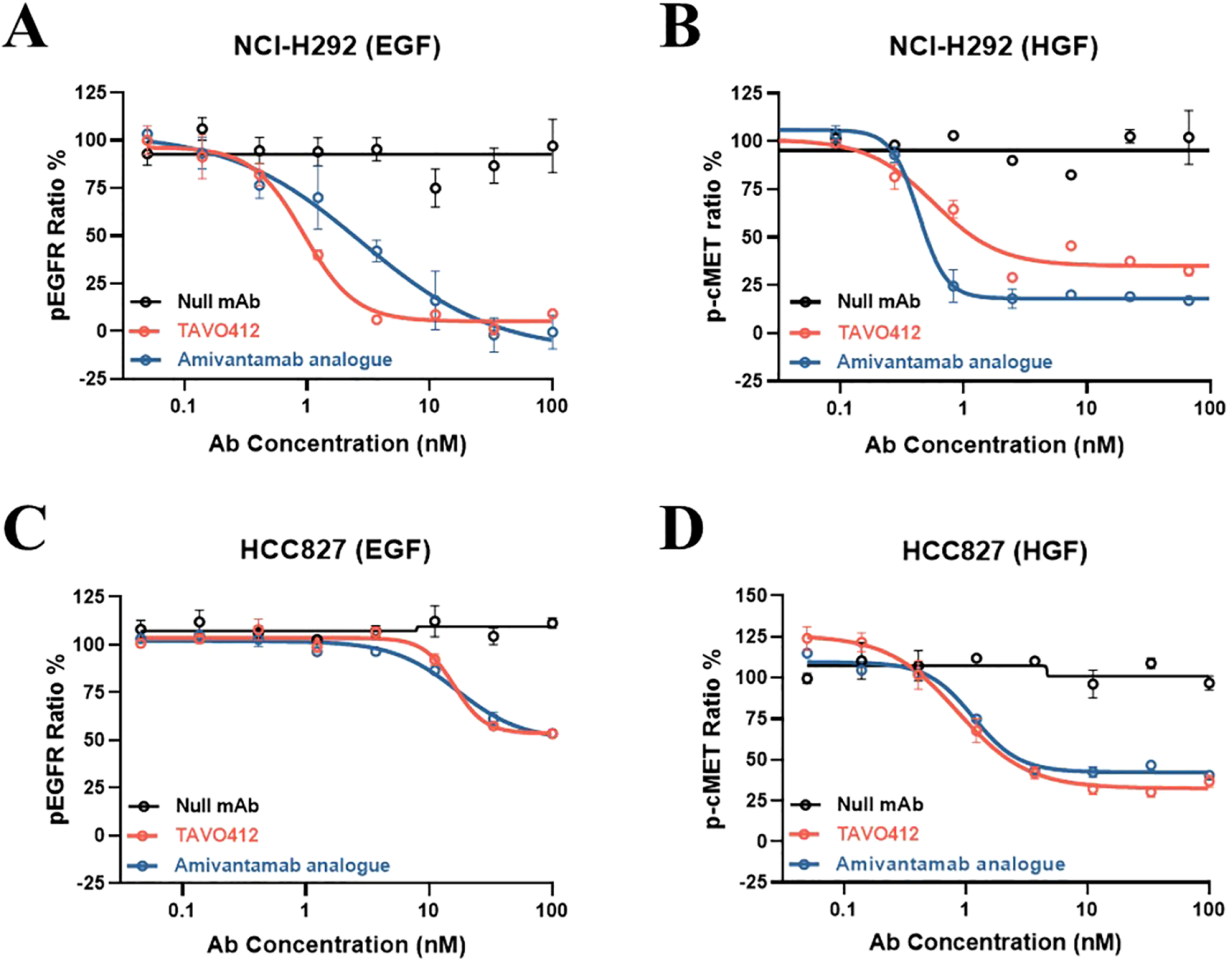
TR-FRET assay demonstration of TAVO412 inhibition of ligand-induced EGFR and cMET phosphorylation in NSCLC cell lines. Inhibition of **(A)** EGF ligand-induced phosphorylation of EGFR and **(B)** HGF ligand-induced phosphorylation of cMET in NCI-H292 cell line. Inhibition of **(C)** EGF ligand-induced phosphorylation of EGFR and **(D)** HGF ligand-induced phosphorylation of cMET in HCC827 cell line. TAVO412 (red open circle), amivantamab analogue (blue open circle) or null mAb (black open circle) were tested. The data from three independent experiments were expressed as the mean ± SEM of duplicate treatments. The amivantamab analogue served as a positive control molecule while the null mAb served as a negative control. The corresponding IC_50_, 95% CI for IC_50_ values and efficacy (span in Y-axis) were reported in **Supplementary Table S4**. The abbreviations were: pEGFR ratio%, phosphorylation rate of EGFR; p-cMET ratio%, phosphorylation rate of cMET; Ab, antibody; nM, nanomolar; EGF, epidermal growth factor; HGF, hepatocyte growth factor; SEM, standard error of the mean.

Patients with hyperactivation of the EGFR and cMET signaling pathways often have higher levels of respective ligand expression (25, 27). Thus, TAVO412 was engineered to inhibit EGF binding to EGFR and HGF binding to cMET; thereby antagonizing paracrine ligand-based EGFR and cMET activations. Using TR-FRET-based assays, TAVO412 was shown to inhibit EGF-induced phosphorylation of EGFR and HGF-induced phosphorylation of c-MET in NCI-H292 cells, whereas wild type *EGFR* and *c-MET* showed minimal baseline EGFR and cMET phosphorylation in the absence of growth factor stimulation (13) (**Figures 2A, B**; **Supplementary Table S4**). TAVO412 inhibited EGF-induced phosphorylation (IC50 of 0.941 nM) with a 3-fold greater potency than that of the amivantamab analogue (IC50 of 2.796 nM). In addition, the amivantamab analogue showed marginally enhanced inhibition of HGF-induced phosphorylation (IC50 of 0.430 nM) when compared to TAVO412 (IC50 of 0.568 nM). (**Figures 2A, B**; **Supplementary Table S4**). The null control antibody did not inhibit receptor phosphorylation. The stronger effect on EGF-induced phosphorylation resulted from blockade of ligand binding and receptor dimerization by the dual epitope EGFR arm as anticipated, while the avidity effect maintained a strong HGF blockade in the tumor cells.

Since HCC827 cells (wild type cMET, and the Ex19Del EGFR mutation which conferred a high baseline level of EGFR phosphorylation) had constitutively activated EGFR, EGF addition did not significantly increase the amount of phospho-EGFR over the high baseline level. Therefore, phospho-EGFR levels remained unchanged by the addition of TAVO412 or amivantamab (**Figure 2C**; **Supplementary Table S4**). Consistent with observations in H292 cells, TAVO412 inhibited HGF-induced cMET phosphorylation in HCC827 (**Figure 2D**; **Supplementary Table S4**).

### TAVO412 had enhanced Fc effector function

Fc effector functions play crucial roles in the efficacy of therapeutic antibodies (28). TAVO412 had enhanced Fc effector functions by incorporating clinically-verified point mutations in the Fc domain (F243L/R292P/Y300L/V305I/P396L) (29). TAVO412 and amivantamab analogue had comparable binding affinities for CD16a, CD32a, and CD64 (manuscript submitted). However, TAVO412 bound to C1q, while the amivantamab analogue and control IgG1 antibodies did not bind to C1q.

**Figure 3:**
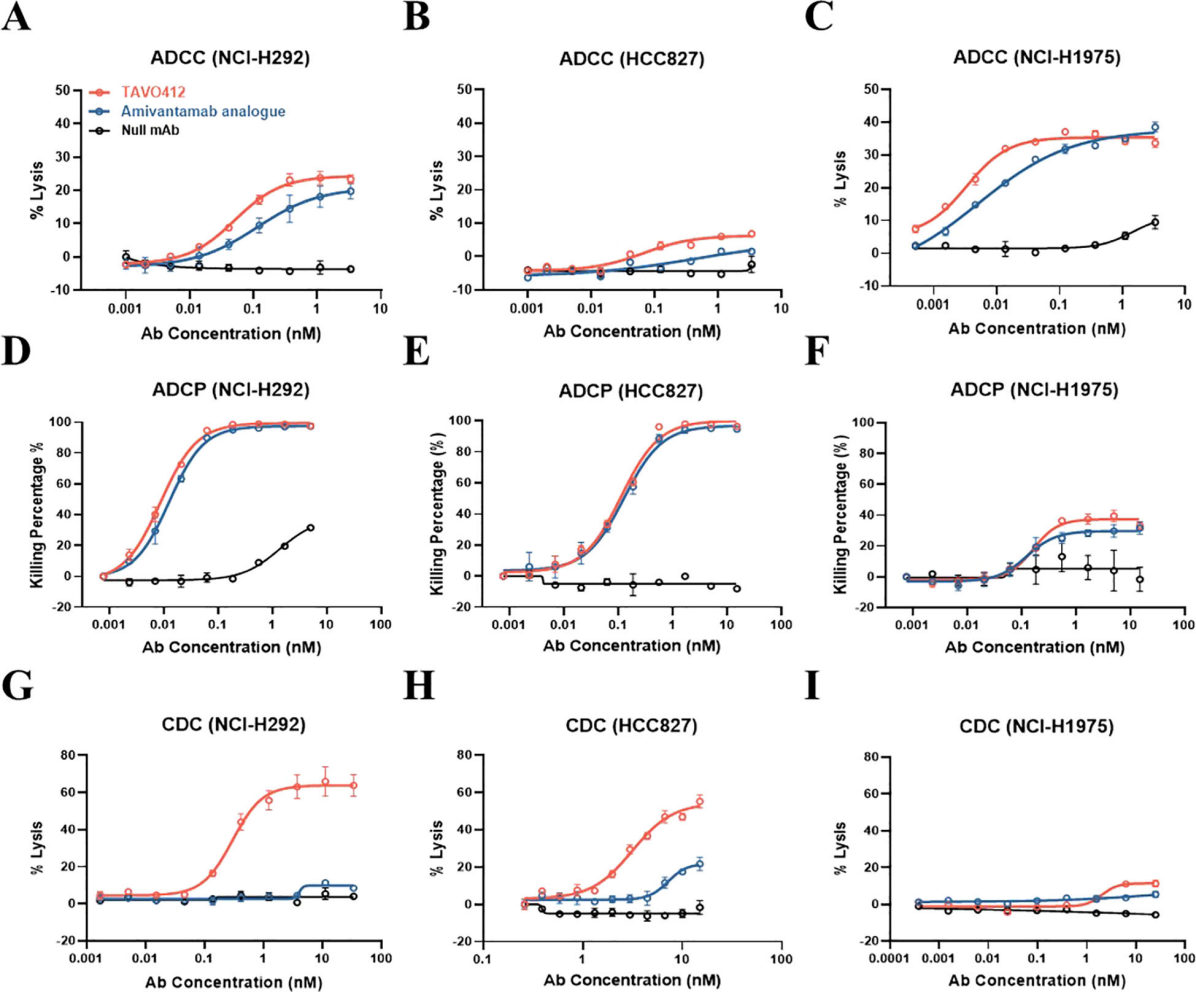
TAVO412 mediated Fc effector functions in NSCLC cell lines. TAVO412 Fc effector function showing ADCC for **(A)** NCI-H292, **(B)** HCC827 and **(C)** NCI-H1975 cells, respectively; TAVO412 Fc effector function showing ADCP for **(D)** NCI-H292, **(E)** HCC827 and **(F)** NCI-H1975 cells, respectively; TAVO412 Fc effector function showing CDC for **(G)** NCI-H292, **(H)** HCC827 and **(I)** NCI-H1975 cells, respectively. TAVO412 (red open circle), Amivantamab analogue (blue open circle) or null mAb (black open circle) were tested. The data from three independent experiments were expressed as the mean ± SEM of duplicate treatments. The amivantamab analogue served as a positive control molecule while the null mAb served as a negative control. The EC_50_, 95% CI for EC_50_ values and efficacy (span in Y-axis) were reported in **Supplementary Table S5**. The abbreviations were: Ab, antibody; nM, nanomolar; ADCC, antibody-dependent cellular cytotoxicity; ADCP, antibody-dependent cellular phagocytosis; CDC, complement-dependent cytotoxicity; SEM, standard error of the mean.

TAVO412’s Fc effector functions were demonstrated in assays that monitored antibody-dependent cellular cytotoxicity (ADCC), antibody-dependent cellular phagocytosis (ADCP), and complement-dependent cytotoxicity (CDC) in NCI-H292, HCC827, and NCI-H1975 cell lines. Although TAVO412 and the amivantamab analogue exhibited comparable binding activity to Fc gamma receptors (FcγR), TAVO412 demonstrated a stronger ADCC response than the amivantamab analogue (NCI-H292: EC_50_ of 0.051 nM versus 0.114 nM; HCC827: 0.066 nM versus 0.483 nM; NCI-H1975: 0.003 nM versus 0.005 nM, for TAVO412 versus amivantamab analogue respectively) (**Figures 3A–C**; **Supplementary Table S5**). Both TAVO412 and amivantamab analogue had similar ADCP responses in the three NSCLC cell lines (NCI-H292: EC_50_ of 0.009 nM versus 0.012 nM; HCC827: 0.112 nM versus 0.126 nM; NCI-H1975: 0.163 nM versus 0.125 nM, for TAVO412 versus amivantamab analogue respectively) (**Figures 3D–F**; **Supplementary Table S5**). Consistent with the ELISA binding to C1q, TAVO412 exhibited CDC killing of NCI-H292 (EC_50_ of 0.297 nM) and HCC827 (3.139 nM), whereas the amivantamab analogue showed only minimal CDC killing (**Figures 3G, H**). With an approximately 10% maximum lysis, H1975 cells were resistant to CDC killing as reported earlier (**Figure 3I**) (30).

To confirm that TAVO412 has enhanced Fc effector functions, TAVO412-A (a TAVO412 isoform without the VEGF binding domain) was compared to TAVO412-A_NF (a TAVO412-A isotype with wild type Fc) and TAVO412-A_SF (a TAVO412-A isoform with a silenced Fc) in the ADCC and ADCP reporter assays, and in CDC induced killing assays using the NCI-H292 cell line. TAVO412-A indeed induced dramatically enhanced ADCC, ADCP and CDC both in terms of potency and efficacy compared to TAVO412-A_NF. The role of the Fc mutations was further confirmed as TAVO412-A_SF had no Fc effector functions (**Supplementary Figures S3A–D**).

Amivantamab Fc effector functions were dependent on cell binding by its anti-EGFR arm (14). To investigate whether TAVO412 had similar EGFR binding-driven Fc effector function, TAVO412-A’s activity was compared to its isoforms with an inert arm in the cMET arm position (EGFR x Inert) or the EGFR arm position (cMET x Inert). In the NCI-H292 cells, EGFR x Inert had comparable Fc effector activities compared to TAVO412-A, while the cMET x Inert did not induce significant responses (**Supplementary Figures S3E–H**). Thus, we demonstrated that TAVO412’s Fc effector functions are also dependent on its binding to EGFR. The EGFR-driven Fc-effector functions were also observed in other cancer cell lines (data not shown).

### TAVO412 suppressed tumor growth in xenograft models with EGFR and cMET degradation

**Figure 4:**
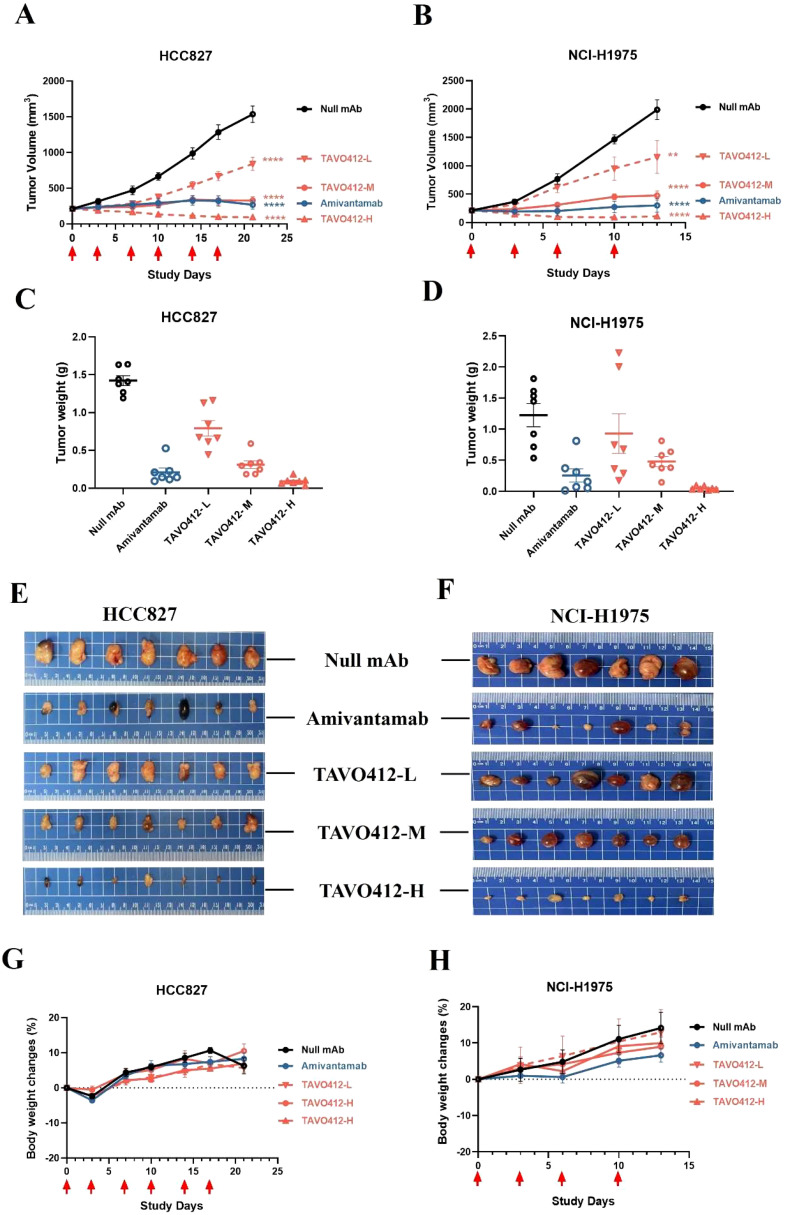
TAVO412 antitumor activity in NSCLC xenograft models. Female Balb/c nude mice bearing NSCLC xenograft tumors were given intraperitoneal injections twice per week of TAVO412 (1, 3, 10 mg/kg), amivantamab analogue (3 mg/kg), or null mAb (10 mg/kg). Tumor growth was monitored twice weekly. The mean tumor growth curves for mice treated with indicated antibodies in **(A)** HCC827 for a total of 6 doses and **(B)** NCI-H1975 for a total of 4 doses were shown. Tumors were collected and tumor weight measured for **(C)** HCC827 at the end of the 21-day observation period and **(D)** NCI-H1975 at the end of the 13-day observation period. Photographs of the resected tumor xenograft specimens at the end of the study were recorded for **(E)** HCC827 tumors and **(F)** NCI-H1975 tumors. The body weight of the tumor-bearing mice treated with indicated antibodies were measured twice weekly until the end of study for **(G)** HCC827 and **(H)** NCI-H1975. The antibodies were labeled: TAVO412 – L for 1 mg/kg dosing (red open down triangle); TAVO412 – M for 3 mg/kg dosing (red open circle); TAVO412 – H for 10 mg/kg dosing (red open up triangle); amivantamab for amivantamab analogue (blue open circle); Null mAb for control (black open circle). The data represented the mean values ± SEM (n = 7/group). The red arrows indicated the specific dosing days. **P < 0.01; ****P < 0.0001; ns: not significant compared to control group. Statistical significance was determined using one-way ANOVA, followed by Tukey’s multiple comparisons test to compare each treatment group with the null mAb group.

The antitumor activities of TAVO412 were assessed in six NSCLC xenograft models that spanned different *EGFR* and *cMET* genotypes, receptor densities, and VEGF-A secretion levels (**Supplementary Table 1**). Monotherapy with TAVO412 at doses of 1 (low), 3 (medium), and 10 (high) mg/kg inhibited tumor growth in both HCC827 and NCI-H1975 xenograft models in a dose-dependent manner (**Figures 4A, B**). The amivantamab analogue administered at 3 mg/kg showed comparable antitumor activities as TAVO412 at similar dosing level (**Figures 4A, B**). Tumor weights and photos taken on the last day of each experiment were consistent with the tumor volume data (**Figures 4C–F**). TAVO412 treatment was well-tolerated without compromising the mice body weight (**Figures 4G, H**). TAVO412 at 10 mg/kg had excellent anti-tumor activity in the other four xenograft models (**Supplementary Figures S4A–H**).

TAVO412 also demonstrated EGFR and cMET receptor degradation *in vivo*. Mice bearing HCC827 or NCI-H1975 tumors were treated with TAVO412 twice. The tumors were collected 24 h after the second dose. The Western blots indicated that the average total protein levels of EGFR and cMET were significantly decreased in both models after TAVO412 treatment; thereby demonstrating receptor degradation (**Supplementary Figures S5A–F**).

To quantify the drug exposure in the animal models, the pharmacokinetics (PK) profile of TAVO412 was studied in the same stain of mice without tumor bearing. Following a single intraperitoneal injection, TAVO412 exhibited linear PK in the dose range from 1 to 3 mg/kg. The half-life was estimated to be 4.6 to 4.8 days (**Supplementary Figure S6**).

### The combination of EGFR TKIs and TAVO412 had stronger anti-tumor efficacy in xenograft models

**Figure 5:**
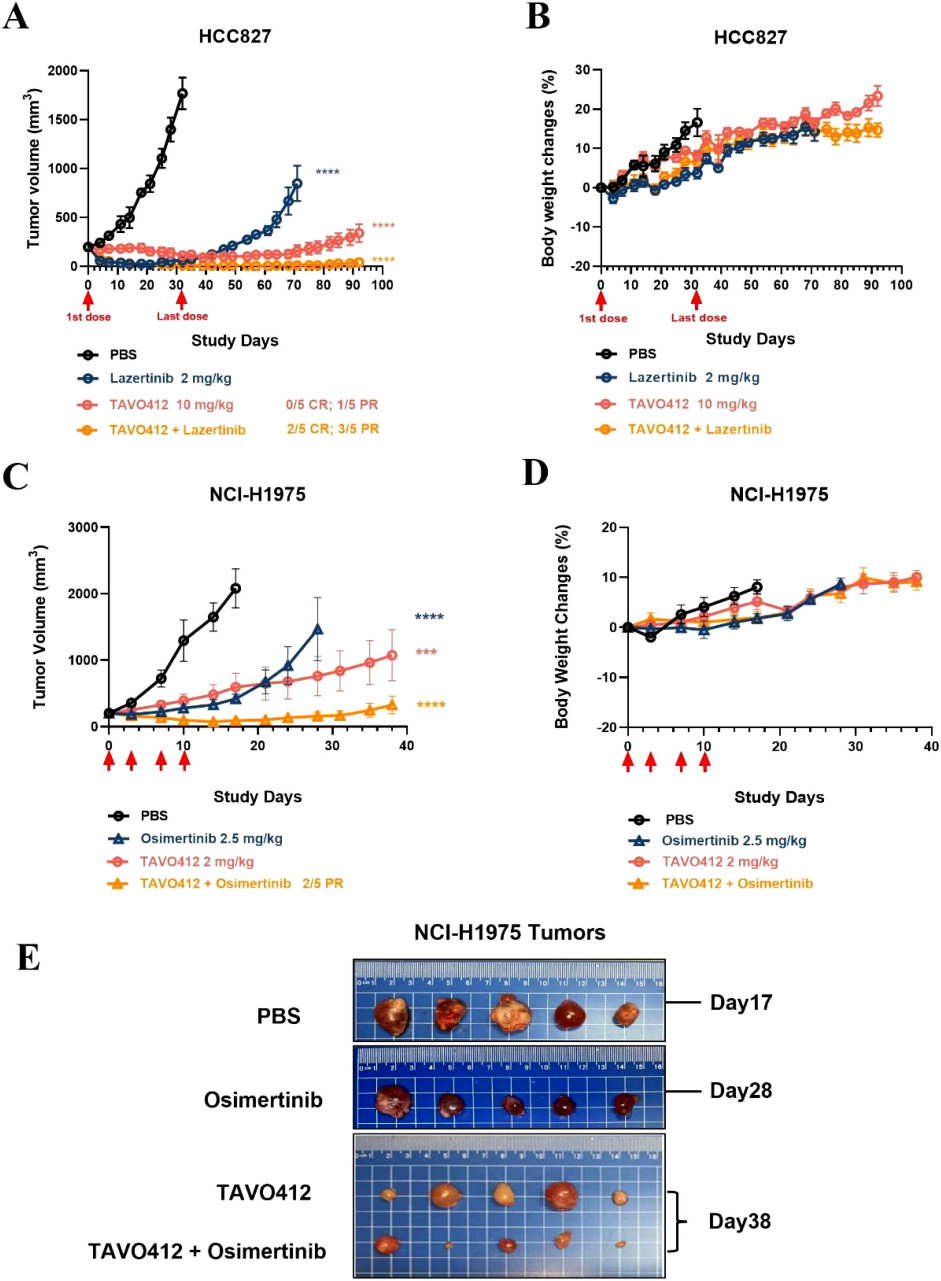
Combination of TAVO412 with Lazertinib and osimertinib in NSCLC xenograft models. Mice bearing HCC827 tumors were treated with TAVO412 (red open circle), Lazertinib (navy open circle), the combination of TAVO412 and Lazertinib (orange open circle) and PBS control (black open circle). TAVO412 was dosed at 10 mg/kg twice per week for a total of 10 intraperitoneal injections (the red arrows indicated the first and last dose). Lazertinib was dosed at 2 mg/kg daily orally for 21 days (day 0 – day 20). **(A)** The HCC827 tumor growth profiles and **(B)** corresponding body weight changes were monitored twice weekly. Mice bearing NCI-H1975 tumors were treated with TAVO412 (red open circle), osimertinib (navy open up triangle), the combination of TAVO412 and Osimertinib (orange open up triangle), and PBS control (black open circle). TAVO412 was dosed at 2 mg/kg twice per week for a total of 4 intraperitoneal injections (the red arrow indicated the specific dosing for TAVO412). Osimertinib was dosed at 2.5 mg/kg daily orally for 14 days (day 0 – day 13). **(C)** The NCI-H1975 tumor growth and **(D)** body weight changes were monitored twice weekly. **(E)** Photographs of the resected tumor xenograft specimens at the end of the study were recorded for NCI-H1975. The treatments were indicated on the left, and the termination day was specified on the right of the photographs. The data represented the mean values ± SEM (n = 5/group). Statistical significance was calculated at day 32 and day 17 for HCC827 and NCI-H1975, respectively by one-way ANOVA followed by Tukey’s multiple comparisons test to compare each treatment group with the null mAb group. ***P < 0.001; ****P < 0.0001. The abbreviations were: ns: not significant compared to control group. CR and PR indicated complete response and partial response; PBS represented phosphate buffered saline; SEM, standard error of the mean.

Amivantamab and lazertinib combination therapy has enhanced antitumor activity in NSCLC patients in clinical trials (31). In light of this, we combined TAVO412 with lazertinib in the HCC827 xenograft model and with osimertinib in the NCI-H1975 xenograft model. Tumor-bearing mice were treated when the average tumor volume reached 200 mm^3^ in the HCC827 model (**Figures 5A, B**). While tumors were effectively inhibited by either single agent or the combination up to day 32, tumor relapses were observed in all the mice treated with lazertinib shortly after the treatment was stopped. TAVO412-treated tumors showed a longer lasting inhibition compared to lazertinib, but tumor regrowth still occurred by the end of the study with only one mouse having partial regression (day 92). The combination of TAVO412 and lazertinib induced complete tumor regression (CR) and partial regression (PR) in 2/5 and 3/5 mice, respectively, until the last observation day (**Figure 5A**). Similarly in NCI-H1975, the combination of TAVO412 with osimertinib showed a significant additive effect: the combination group having a significantly smaller tumor burden compared to each monotherapy group and the control (**Figures 5C, E**). NCI-H1975 tumors treated with TAVO412 appeared paler compared to tumors in other treatment groups (**Figure 5E**), which indicated reduced vasculature due to VEGF-A neutralization by TAVO412. This phenomenon was also observed in other xenograft models with different tumor cell types (data not shown). Treatments of TAVO412 were well tolerated with no body weight loss (**Figures 5B, D**). These findings showed that the combination of TAVO412 with either lazertinib or osimertinib had stronger tumor growth inhibition compared to the monotherapies and prevented relapse in the mouse models of HCC827 and NCI-H1975. We used 2 mg/kg TAVO412 (suboptimal dose) in NCI-H1975 models to assess combination potential, and 10 mg/kg (optimal dose) in HCC827 models to demonstrate complete tumor control. This dual approach allowed evaluation of both combination effects and maximal efficacy.

### EGFR TKIs enhanced the efficacy of TAVO412 by stabilizing EGFR receptor on cell surface or enhancing ADCC

**Figure 6:**
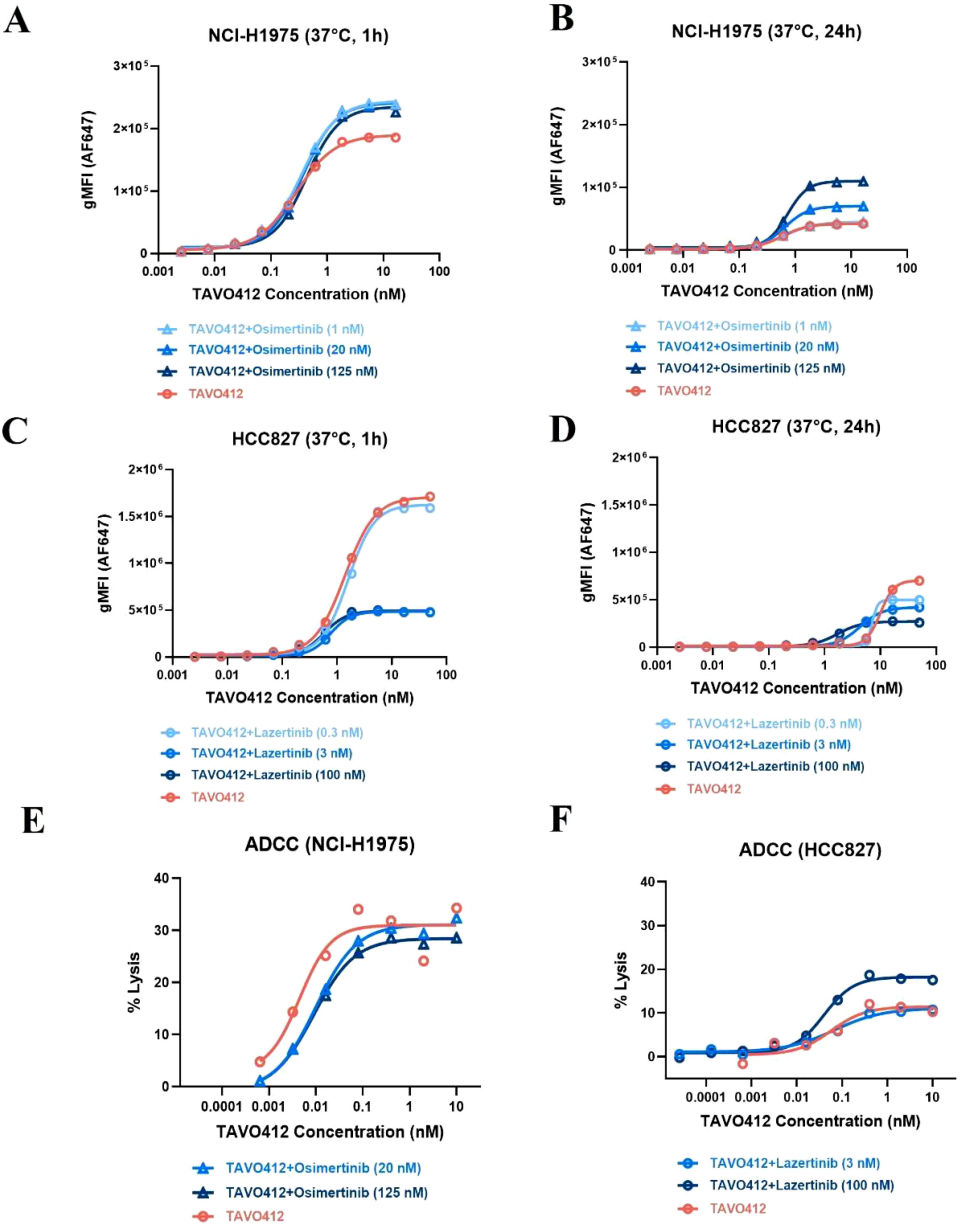
EGFR-TKI affected the cell binding and TAVO412 ADCC effector function. **(A, B)** NCI-H1975 and **(C, D)** HCC827 cells were pre-treated with osimertinib and lazertinib, respectively, for 48 h at specified concentrations. Then TAVO412 was added to the cells in a serial dilution and incubated for 1 h **(A, C)** or 24 h **(B, D)** at 37°C. The binding of TAVO412 in both cell lines were measured by flow cytometry. The EC_50_, 95% CI for EC_50_ values and efficacy (span in y axis) were reported in **Supplementary Table S6**. **(E)** NCI-H1975 and **(F)** HCC827 cells were pre-treated with Osimertinib and Lazertinib, respectively, for 48 h at specified concentrations and then ADCC-induced killing was assessed. The treatments were labeled: TAVO412 alone: red open circle; TAVO412 + osimertinib: light, medium and dark blue open up triangle for addition of 1, 20 and 125 nM of osimertinib, respectively; TAVO412 + lazertinib: light, medium and dark blue open circle for addition of 0.3, 3 and 100 nM of lazertinib, respectively; Representative data from 2 to 3 independent experiments are shown. The EC_50_, 95% CI for EC_50_ values and efficacy (span in y axis) were reported in **Supplementary Table S7**. The abbreviations were: gMFI, geometric mean fluorescent intensity; h, hour; nM, nanomolar; ADCC, antibody-dependent cellular cytotoxicity; SEM, standard error of the mean.

The basis for the EGFR-TKI enhancement of TAVO412 was further probed by conducting cell-based assays. H1975 cells were treated with varying concentrations of osimertinib for 48h at 37°C and then stained with either anti-EGFR or anti-cMET detection antibodies to assess receptor density via flow cytometry analysis. When compared to the absence of osimertinib treatment, the incubation of H1975 with osimertinib at 0.1, 1, and 20 nM resulted in 1.1-, 1.5-, and 2.6-fold MFI increase in EGFR binding but did not affect cMET binding levels (**Supplementary Figures S7A, B**). The binding of TAVO412 to osimertinib-treated H1975 cells was then evaluated at both 1h and 24h at 37°C. The level of TAVO412 binding (at 16 nM TAVO412 for all the following comparisons) after 24h was 80% lower than the binding after 1h (**Figures 6A, B**). The longer incubation time for binding could manifest in higher levels of internalization and degradation of the receptors. At 1 h, the MFI ratios of TAVO412 binding to H1975 cells treated with osimertinib at 1 nM, 20 nM, or 125 nM to that without osimertinib treatment were 1.3, 1.3, and 1.2, respectively. After 24h, these MFI ratios were 1.1, 1.7, and 2.6, respectively, indicating that there was an osimertinib dose-dependent increase in TAVO412 binding. However, the TAVO412 increase in cell binding did not further increase the ADCC effect *in vitro* (**Figure 6E**).

Since lazertinib showed synergy with TAVO412 *in vivo* in the HCC827 model, we assessed the impact of lazertinib on receptor densities, TAVO412 binding, and ADCC effects on HCC827 cells. In comparison to the absence of lazertinib treatment, culturing HCC827 cells with lazertinib at 0.3, 3, and 100 nM at 37°C after 48 h led to 10, 60 and 70% reductions in cMET receptor density, respectively. Surprisingly, the EGFR density levels were unaffected (**Supplementary Figures S7C, D**). As with H1975 cells, TAVO412 alone demonstrated decreased binding to HCC827 cells (by 60%, at 16 nM TAVO412 for all the following comparisons) after 24 h versus 1 h at 37°C (**Figures 6C, D**). Upon addition of 0.3, 3, and 100 nM lazertinib, TAVO412 binding was decreased by 0, 70, and 70% at 1 h, respectively. After 24 h, the TAVO412 binding to HCC827 was decreased by 20, 30 and 60%, respectively (**Figures 6C, D**). This reduction in TAVO412 binding could be associated with the decreased cMET receptor density upon lazertinib treatment. HCC827 cells exhibited a 14-fold higher TAVO412 binding (at 16 nM) than on H1975 after 24 h (**Figures 6B, D**), which corresponded to the relative receptor densities of EGFR and cMET (**Supplementary Table S1**). There was still a 2.5-fold higher binding of TAVO412 to HCC827 cells compared to H1975 cells with EGFR-TKI supplemented (100 nM lazertinib versus 125 nM osimertinib; **Figures 6B, D**). Despite a reduced binding of TAVO412, the vitro ADCC assay showed an enhancement of the killing effect by addition of lazertinib at 100 nM concentration (**Figure 6F**).

### Combination of TAVO412 with conventional chemotherapy and radiotherapy induced complete regression of tumors *in vivo*

**Figure 7:**
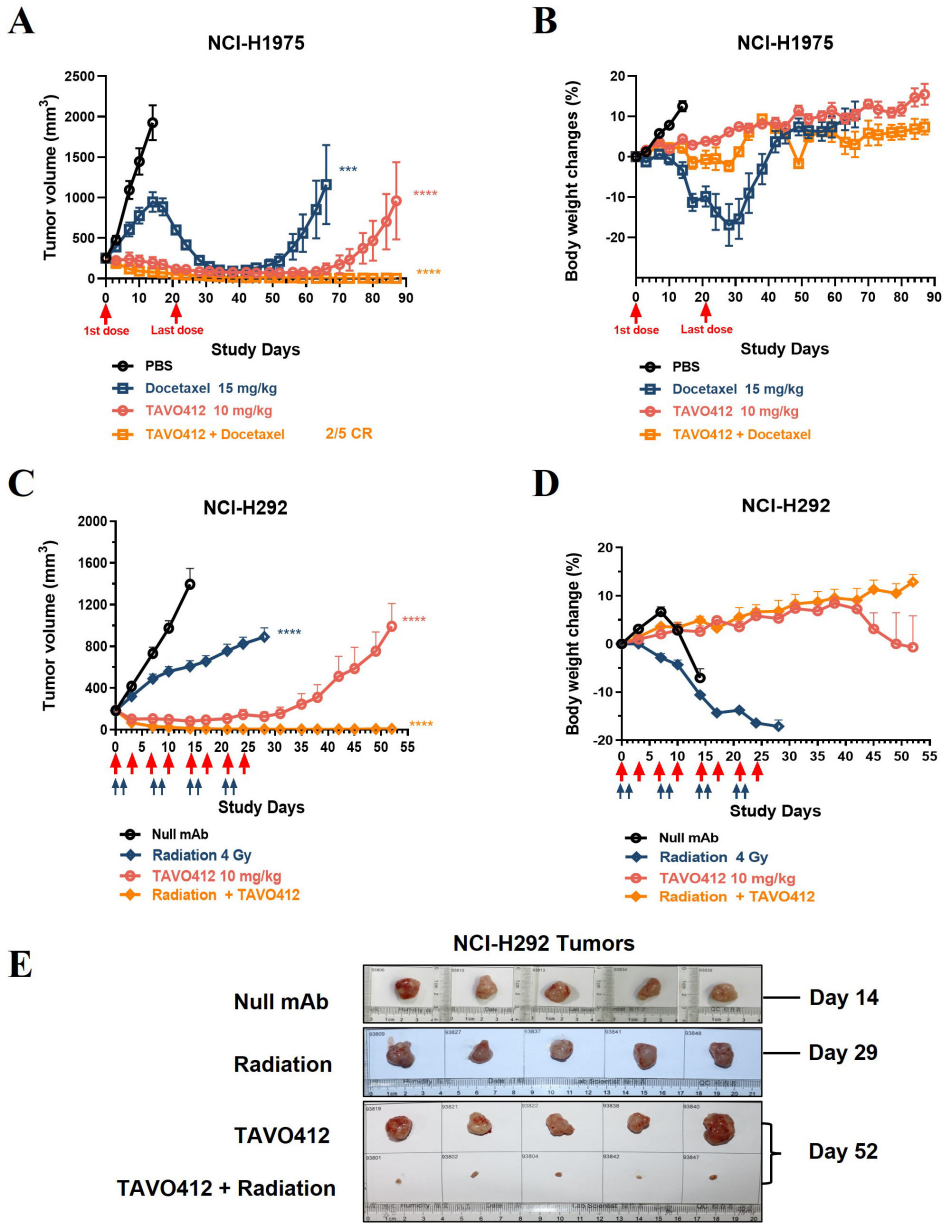
Treatment with TAVO412 in combination with docetaxel or radiation induced tumor regression in NSCLC xenograft models. **(A, B)** Mice bearing NCI-H1975 tumors were given intraperitoneal injections twice per week with: TAVO412 (10 mg/kg, red open circle), Docetaxel (15 mg/kg, navy open square), the combination of TAVO412 and Docetaxel (orange open square), or PBS vehicle control (black open circle). The red arrows mark the first and last dosing with a total of 8 intraperitoneal injections administered over this period. The mice were monitored 2X/week for **(A)** tumor growth and **(B)** body weight changes. **(C-E)** Mice bearing NCI-H292 tumors were treated with: TAVO412 (10 mg/kg, right open circle), x-ray radiation (4 Gy per fraction, navy open diamond), the combination of TAVO412 and irradiation (orange open diamond), or null mAb (10 mg/kg, black open circle). The red arrows marked the dosing days for TAVO412 and the light blue arrows marked the irradiation treatment days. The mice were monitored twice weekly for **(C)** tumor growth and **(D)** body weight changes. **(E)** Photographs of resected NCI-292 xenograft tumors collected on the termination days indicated to the right of figure. The treatments listed on the left. The data represent the mean values ± SEM (n = 5/group). Statistical significance was calculated at day 14 for both models by one-way ANOVA followed by Tukey’s multiple comparisons test to compare each treatment group with the null mAb group. ***P < 0.001; ****P<0.0001. The abbreviations were: ns, not significant compared to control group; Gy, gray; CR indicates complete response.

We examined whether combining TAVO412 with either taxane chemotherapy or radiotherapy would yield a more effective anti-cancer treatment. Chemotherapy and radiotherapy are extensively used in the clinic to treat NSCLC patients. Docetaxel was selected to combine with TAVO412 to investigate their combination effect on NCI-H1975 tumor growth in xenograft mouse models (**Figures 7A, B**). Both TAVO412 and docetaxel were dosed twice a week for 4 weeks, via intraperitoneal injection. Initially, docetaxel as a single agent showed minimal tumor control effect. However, all tumors in this group reached their peak volume and began to regress at day 14. The maximal tumor control effect was observed at day 38 (14 days after the end of treatment), and then 3 out of 5 tumors began to regrow and 2 out of 5 tumors remained stable with a volume of less than 20 mm^3^ by the end of the study (day 87) (**Figure 7A**). TAVO412 exhibited a strong tumor growth inhibition effect in this model as a monotherapy, with 4 out of 5 mice achieving partial regression by the end of the treatment period (day 28). After cessation of treatment, 3 out of 5 tumors gradually regrew, while 2 out of 5 tumors remained stable by the end of the study. The combination of TAVO412 with docetaxel demonstrated superior antitumor activity compared to each single agent, with 2 out of 5 mice achieved complete regression, and 3 out of 5 mice achieved partial regression with a tumor volume measured less than 10 mm^3^ at the end of the study.

The NCI-H292 xenograft model was used to examine the effect of TAVO412 and X-ray irradiation combination therapy based on published evidence demonstrating that the anti-EGFR antibody nimotuzumab potentiates radiation sensitivity more effectively in this cell line than in NCI-H1975 (32). NCI-H292 tumor cells were implanted in immune-compromised mice and the treatments were started when the tumor volume reached ~200 mm^3^. TAVO412 was dosed twice a week for 4 weeks by intraperitoneal injection, irradiation (4 Gy per fraction) was performed on the first two days each week for 4 weeks in total, while the combination group followed the same regimen with each monotherapy (**Figures 7C–E**). The irradiation therapy alone only produced modest antitumor activity; all the tumors continued to grow, albeit at a slower rate compared to null control-treated tumors. TAVO412 alone showed a more potent tumor control effect with NCI-H292 tumors: all the tumors regressed to a tumor volume below the starting size, and 2/5 mice achieved partial regression by day 28. However, all the tumors gradually regrew when the treatment stopped. In contrast, the combination treatment induced a remarkable decrease of tumor burden: all the tumors regressed quickly starting from the beginning of treatment (the tumor size regressed to less than 10 mm^3^ by day 21), and the effect persisted in all treated animals until the end of the study (**Figure 7C**). **Figure 7E** showed photographs of resected xenograft tumors taken at the endpoint of each group and the specimen sizes were consistent with the tumor volume data (**Figure 7C**). As anticipated, both Docetaxel and X-ray irradiation resulted in weight loss in mice. Notably, mice in the TAVO412 combination group did not experience weight loss. Instead, they showed a consistent increase in body weight throughout the entire study period (**Figures 7B, D**).

## Discussion

Although the overall mortality rate of NSCLC has decreased due to the identification of disease-specific oncogenes coupled with personalized, genotype-directed therapies, the 5-year survival rate remains poor at 17.4%. In relapsed patients, drug resistance develops through emergence of secondary mutations, activation of by-pass signaling pathways, or phenotypic transformation. The development of novel therapies that can overcome such diverse resistance mechanisms remains a substantial clinical need. EGFR, cMET, and VEGF play critical and complementary roles in NSCLC cell survival, proliferation, and resistance to conventional therapies (33). Hence, we developed TAVO412, a single trispecific antibody-based molecule that inhibited EGFR, cMET, and VEGF-A. The molecular construct of TAVO412 was designed with a dual epitope variable heavy-chain only (VHO) EGFR binding arm on the N-terminal and an anti-VEGF-A ScFv domain on the C-terminal of one heavy chain, and an anti-cMET Fab arm on the other chain. Utilizing Knob-in-Hole mutations, TAVO412 was expressed in a single CHO cell line. Its developability characteristics and downstream processing were comparable to monoclonal antibodies in general, with high-yield production (2.5 g/L in CHO cells), stable monomeric purity (>97%), high thermal stability (Tm > 65°C), and no post-translational modification mutation hotspots in the CDR region. TAVO412 binds human targets at high affinities; it is fully cross reactive with the monkey targets, but not those of the mouse (data not shown, is published elsewhere). We demonstrated how TAVO412 manifested multiple mechanisms of action, including ligand blocking (**Figures 1D, E**), receptor phosphorylation inhibition (**Figure 2**), Fc effector functions including ADCC, ADCP, and CDC (**Figure 3**) and EGFR/cMET receptor degradation (**Supplementary Figure S5**). All of these mechanisms of action translated into excellent tumor growth inhibition effects *in vivo*, as observed in a panel of NSCLC xenograft models with diverse receptor density and EGFR and kRAS mutation status (**Figure 4**; **Supplementary Table 1**; **Supplementary Figure S4**).

Receptor degradation was observed in tumor samples for both EGFR and cMET 24 h after two doses of TAVO412, as compared to control tumors (**Supplementary Figure S5**). Such a mechanism could remove dysfunctional autocrine signaling of EGFR and cMET. The effect of receptor degradation by TAVO412 could be linked to immune effector-based mechanisms, such as trogocytosis (antibody-dependent cellular trogocytosis, ADCT), which had been identified as a dominant mechanism of antibody-directed receptor downregulation and tumor cell killing *in vivo* for amivantamab (34). However, further studies are needed to confirm this hypothesis.

TAVO412 was capable of mediating ADCC, ADCP, and CDC-induced killing effects, while amivantamab only showed ADCC and ADCP effects, and not CDC (14, 34). An earlier published study (35) showed that zanidatamab, an anti-HER2 biparatopic antibody, could enhance CDC by enhancing receptor clustering, and the effects were correlated with the receptor densities. We obtained similar experimental results showing that the dual epitope EGFR VHO promotes antibody clustering on the cell surface more effectively than any of the monovalent or bivalent parental antibodies (manuscript already submitted). TAVO412 was designed with dual epitope EGFR plus cMET bindings to enhance its cell surface presentation and clustering in addition to its enhanced Fc functions, which resulted in much stronger effector functions.

We used Balb/c Nude mice for *in vivo* xenograft model to test the anti-tumor effects of TAVO412 and comparator molecules. These mice are characterized by a mutation in the Foxn1 gene, leading to an absent or underdeveloped thymus and, consequently, a deficiency in T-cell production. However, the nude mice maintain an active macrophage system and exhibit high levels of NK cell reactivity (36).Therefore, the ADCC and ADCP effects can be tested with mouse NK cells (37).

The HCC827 cell line, with the *EGFR* Ex19Del mutation that accounts for ~ 60% of *EGFR* mutations in lung cancer, is highly responsive to first-generation EGFR TKIs, such as erlotinib. The NCI-H1975 cell line harbors both the L858R and the T790M mutations that confer resistance to first- and second-generation TKIs; but retains sensitivity to third - generation TKIs such as osimertinib. The remaining four cell lines (NCI-H460, NCI-H1299, NCI-H358 and NCI-H596) have wild-type *EGFR* and *cMET* genotypes at different receptor density levels. The NSCLC model NCI-H1975 (L858R/T790M) and HCC827 (Ex19Del) are sensitive to TKIs and amivantamab. In both models, TAVO412 exhibited potent and comparable antitumor activity to the amivantamab analogue (**Figure 4**). TAVO412 demonstrated anti-tumor activities against four NSCLC xenograft models with low, moderate, and high levels of wild-type genotype *EGFR* expression. (**Supplementary Table S1**; **Supplementary Figure S4**). TAVO412 and the amivantamab analogue were equally potent in inhibiting the growth of NCI-H358 and NCI-H596 xenografts, while only TAVO412 produced moderate antitumor activity in NCI-H460 and NCI-H1299 xenografts. Amivantamab showed no antitumor activity in these two models. The difference in response to TAVO412 could be explained by the different levels of EGFR receptor expression in these cell lines, considering the fact that Fc-dependent activity plays a critical role in tumor inhibition efficacy *in vivo* while the effector functions were driven by the anti-EGFR arm of TAVO412. Indeed, the two models with moderate responses had low EGFR and cMET expression levels (**Supplementary Table S1**). The fact that the antigen density on tumor cells must exceed a threshold for even high-affinity IgG antibodies to mediate ADCC could explain why both the amivantamab analogue in our study and cetuximab in another study did not show any antitumor activity in NCI-H460 xenografts (38, 39). Nonetheless, TAVO412 was more efficacious than the amivantamab analogue and cetuximab in these two low EGFR/cMET density models. TAVO412 had stronger tumor growth inhibition than amivantamab and cetuximab, since the design had more engineered MOA that included: angiogenesis control by the anti-VEGF arm; enhanced ADCC, ADCP, and CDC mediated by the unique dual-epitope EGFR and cMET binding epitopes. These results suggested that TAVO412 could be an effective treatment option for a broad range of NSCLC patients, regardless of the EGFR or KRAS mutational status.

Considering that many patients could have been treated with standard of care treatments, we explored the potential role for TAVO412 as a combination partner. Here, we demonstrated how TAVO412 showed substantially greater antitumor activity when combined with either third-generation TKIs or conventional chemoradiotherapy, compared to single-agent treatments, and prevented the emergence of resistance.

We observed enhanced TAVO412 anti-tumor effects when tumors were treated in combination with EGFR-TKIs in several *in vivo* models. To explore possible underlying mechanisms, we conducted a series of *in vitro* cell-based assays that showed how osimertinib increased EGFR receptor density on NCI-H1975 cell surface, resulting in an enhanced TAVO412 cell binding (**Supplementary Figures S7A, B**; **Figures 6A, B**). Similarly, erlotinib induced higher levels of cetuximab binding to EGFR on NSCLC tumor cells, which translated to enhanced cytotoxicity and a stronger *in vivo* anti-cancer effect (40). We corroborated their conclusions by demonstrating that osimertinib, a third-generation EGFR-TKI, exerted similar EGFR receptor stabilizing effects in NCI-H1975 cell line (with *EGFR* mutations) and led to enhanced TAVO412 cell binding. Other reports showed that while EGFR-TKIs were capable of decreasing EGF-induced internalization and could promote EGFR dimerization in a ligand-independent manner (41–44). While the osimertinib-enhanced TAVO412 binding on the cell surface did not translate into enhanced ADCC activity, the induction of stronger tumor growth inhibition could be linked to other mechanisms involving stronger blockade of signal transduction, enhanced ADCP, and CDC.

Amivantamab in combination with lazertinib in the CHRYSALIS-2 (NCT04077463) clinical trial has demonstrated a clinical benefit rate (CBR) of 57% in patients with common *EGFR* exon 19 deletion or L858R mutations who had previously progressed on osimertinib and platinum-based chemotherapy (31). Likewise, we confirmed that TAVO412 with lazertinib demonstrated enhanced tumor growth inhibition effects *in vivo*. However, the combination of TAVO412 with lazertinib in treating HCC827 cells showed more interesting mechanistic aspects. Lazertinib treatment reduced the cMET receptor density in a dose-dependent manner without an apparent impact on the EGFR density. Perhaps some crosstalk between the two receptors and signal pathways could be associated with the reduced levels of TAVO412 binding at physiological conditions (**Supplementary Figures S7C-D**; **Figures 6C, D**). Nonetheless, lazertinib showed a trend of enhancing the ADCC killing effect of TAVO412 (**Figure 6F**).

The correlations of cell surface drug presentation with effector functions could involve a dynamic balance among extracellular EGFR and cMET receptor densities, receptor internalization/degradation rates, and recycling. Our *in vitro* results only provided a glimpse of the complexities. Moreover, drug presentation was only one of several factors that could influence effector functions. An example from our study highlighted that despite HCC827 having significantly higher TAVO412 presentation than H1975, it exhibited a weaker ADCC effect (**Figures 3B, C**, **7E, F**). The balance amongst the activating and inhibitory signaling pathway ultimately determines effector cell responses. For instance, the density of ULBP1 ligands on NSCLC tumor cells could affect NKG2D regulation of NK cell induced-killing (45).

Chemotherapy and radiotherapy are the cornerstone of combined-modality therapy used to cure early and locally advanced NSCLC patients (46). The activation of EGFR, cMET, and VEGF signaling pathways has been linked to chemotherapy and radiotherapy resistance (25, 47). Blocking these pathways has been shown to enhance the effectiveness of chemotherapy and radiotherapy in preclinical studies (38, 48–50). However, in clinical trials, the combination of chemoradiotherapies (CRT) with anti-EGFR or anti-VEGF agents has often been disappointing because of the lack of significant improvement in survival. Additionally, there is a risk of excessive toxicity when combining CRT with targeted agents (9, 47, 51). Our studies provided proof of concept demonstration of the anti-tumor response of chemo- or radio-therapy when combined with TAVO412. In these models, the mice that received either docetaxel or radiation alone experienced a body weight loss of approximately 20%, while mice that received combination treatments maintained their normal growths. (**Figures 7B, D**). Thus, TAVO412 could have a protective effect against radiation and docetaxel treatment in animal models. Notwithstanding, careful study design and proper patient selection are necessary for further clinical studies to ensure both safety and efficacy when combining TAVO412 with CRT.

Besides the NSCLC tumor models, TAVO412 was also tested in several other tumor models including triple negative breast, gastric, esophageal, head and neck cancers, and pancreatic ductal adenocarcinoma. Manuscripts reporting the results have been prepared and will be published elsewhere separately (37). Although we did not conduct a standalone study to assess the individual contributions of the EGFR, cMET, and VEGFA arms in one NSCLC xenograft model using inert arm comparator molecules, we performed such study in a triple-negative breast cancer (TNBC) model. The results showed that, while TAVO412 has a monovalent anti-soluble VEGF-A arm with a slightly weaker binding affinity than bevacizumab, it demonstrated significantly stronger tumor growth inhibition in the model compared to bevacizumab or bevacizumab plus amivantamab at comparable dose levels (data submitted in another manuscript on TNBC). The anti-VEGF effect driven by EGFR/cMET targeting exhibited stronger and more promising anti-tumor results. We also anticipate that TAVO412 will have fewer anti-VEGF-related toxicity issues compared to molecules lacking homing mechanisms. In conclusion, TAVO412, which targeted EGFR, cMET, and VEGF, was demonstrated to have multiple mechanisms of antitumor activity in multiple preclinical models with varying levels of EGFR, cMET, and VEGF. TAVO412 showed potential as a valuable agent for combination therapy with standard-of-care treatments. This combination approach could potentially delay or prevent the development of drug resistance, providing valuable therapeutic options for lung cancer patients. While preparing this manuscript, TAVO412 has been tested in a Phase 1a clinical trial (NCT06761651) and has demonstrated reasonable safety, tolerability, and preliminary positive efficacy signals in NSCLC and other tumor types.

## Materials and methods

### Test antibodies and reagents

TAVO412 was a trispecific antibody with F243L/R292P/Y300L/V305I/P396L and Knob-in-Hole mutations. The molecular construct was designed with dual EGFR binding domains on the N-terminal and anti-VEGFA ScFv on the C-terminal of one heavy chain, and an anti-cMET Fab arm on the other chain. TAVO412 was expressed from a single stably-transfected CHO cell line, purified using Protein A and ion exchange chromatography, and characterized by SEC-HPLC and SDS-PAGE. Thermal stability was evaluated by differential scanning fluorometry (DSF) with a 1°C/min ramp (20–100°C). The amivantamab analogue (sequences referred to World Health Organization Proposed INN List 121) was generated in-house (52). Anti-gp120 hIgG1 was produced in-house to serve as a negative control antibody (null mAb) in cell-based experiments. HIgG1 (HAOKESAIYE, Beijing) served as an isotype control antibody (null mAb) in the animal studies. Lazertinib (Selleck #S8724), osimertinib (MCE LLC #HY-15772A), and docetaxel (injection from the Yangtze River Pharmaceutical Group, Jiangsu, China) were used in the combination experiments.

### Tumor cell lines

NCI-H460, NCI-H1299, NCI-H358 and NCI-H596 cell lines (ATCC); and NCI-H1975, HCC827 and NCI-H292 cell lines (National Collection of Authenticated Cell cultures, Shanghai, China) were authenticated using short tandem repeat profiling and screened for mycoplasma contamination using the Myco-Lumi™ Luminescent Mycoplasma Detection Kit (Beyotime, #C0297M). Cells were cultured following ATCC cell line-specific recommendations. Frozen human peripheral blood mononuclear cells (PBMCs) were purchased from ALLCELLS and SAILYBIO.

### Binding to EGFR and cMET expressing NSCLC cells

The NSCLC cells were seeded at a density of 50,000 cells per well in 96-well plates and treated with the test articles. After 1 h incubation at 4°C, the cells underwent three washes with fluorescence activated cell sorting (FACS) buffer (PBS supplemented with 2% (v/v) fetal bovine serum). The cells were then incubated with AF647 goat anti-human IgG1 Fc (Jackson ImmunoResearch, 109-605-190) in the dark for 30 min at 4°C, washed three times with FACS buffer, and resuspended in FACS buffer for flow cytometry (Beckman CytoFLEX) experiments. The cells were gated initially based on forward and side scatter (FSC vs SSC) to eliminate debris and to define a population gate (P1). P1 was then analyzed on forward scatter height (FSC-H) versus forward scatter area (FSC-A) to isolate single cells (P2). The geometric mean fluorescence intensity (gMFI) of P2 cells was calculated with the CytExpert 2.4 software (Beckman Coulter). The gMFI values on the y axis was plotted against the antibody concentration on the x axis using a four-parameter logistic (4PL) model. EC_50_ values, efficacy (Y-axis span), and 95% confidence intervals (CI) were calculated in GraphPad Prism 9.3.1(GraphPad Software, Inc.).

To assess the effect of EGFR-TKIs to TAVO412 binding to tumor cells, NCI-H1975 and HCC827 cells were pre-treated with osimertinib or lazertinib for 48 h at 37°C. The cells were then cocultured with TAVO412 for 1 h and 24 h respectively at 37°C, and the gMFI level of AF647 goat anti-human IgG1 Fc was measured by flow cytometry as stated above.

### Competitive ligand binding in HCC827 cells

Upon plating 50,000 HCC827 cells per well in 96-well plates, TAVO412, amivantamab analogue, or Null mAb were added in FACS buffer. After incubation 1 h at 4°C, the cell-antibody mixtures were washed three times with the FACS buffer. Either 50 μL of 1 μg/mL EGF or 50 μL of 0.2 μg/mL Biotin-HGF were added; incubated in the dark at 4°C for 1 h; and then followed by three FACS buffer washes. Rabbit anti-human EGF antibody (Sino Biological, #10605-T16) and Alexa Fluor 488 (AF488)-labeled anti-rabbit IgG1 (Jackson ImmunoResearch, #111-545-144) were added to test for EGF binding, while AF488 streptavidin (Invitrogen, #S11223) was added to test HGF binding. After 0.5 h incubation at 4°C, the cells were washed three times and resuspended in FACS buffer for flow cytometry analysis (Beckman CytoFLEX) with the FACS gating strategy as described above. The AF488 fluorescence signals of the P2-gated cells were captured and the gMFI was calculated with CytExpert 2.4 software (Beckman Coulter). The gMFI of the cells was plotted on the y axis against the antibody concentration on the x axis using a four-parameter logistic (4PL) model. IC_50_ values, efficacy (y-axis span) and 95% CI for IC_50_ were calculated in GraphPad Prism 9.3.1(GraphPad Software, Inc.).

### Inhibition of ligand-induced receptor phosphorylation

Time-Resolved Fluorescence and Resonance Energy Transfer (TR-FRET) assay was used to measure the phosphorylation of EGFR and cMET receptors in NSCLC cells. Forty thousand cells were seeded in RPMI 1640 medium per well in 96-well plates overnight and starved for 24 h, before being treated with test articles for 1 h at 37°C. After stimulation with 22 nM EGF or 10 nM HGF at 37°C for 5 and 15 min respectively, the cells were lysed, and the levels of receptor phosphorylation were monitored using TR-FRET kits (Bioauxilium, KIT-EGFRP-5000 or KIT-METP-5000). The phosphorylation rate (%) on the y axis was plotted against the antibody concentration on the x axis using a four-parameter logistic (4PL) model. The IC_50_, efficacy (y-axis span), and 95% CI for IC_50_ values were calculated in GraphPad Prism 9.3.1 (GraphPad Software, Inc.). The phosphorylation rate (%) was determined by calculating [(Signal _Test article_-Signal _detection buffer control_)/(Signal _None treated_- Signal _detection buffer control_)] ×100%.

### ADCC assays

Primary ADCC killing assays assessed *in vitro* killing of NSCLC cells. Ten thousand tumor cells were plated per well in RPMI1640. Antibodies were added to the wells and incubated at 37°C for 15 min. Upon thawing, 500,000 PBMCs in RPMI1640 were added to each well and incubated at 37°C for 4 h. The release of lactate dehydrogenase (LDH) was measured using a Roche Cytotoxicity Detection Kit. Controls included untreated effector and target cells, target cells only, and target cells plus 0.2% (w/v) Triton X-100. The lysis ratio on the y axis was plotted against the antibody concentration on the x axis using a four-parameter logistic (4PL) model. EC_50_ values, efficacy (y-axis span) and 95% CI for EC_50_ were calculated in GraphPad Prism 9.3.1(GraphPad Software, Inc.). Lysis ratio (%) was determined by the following equation with OD values (492 nm - 650 nm):


$$\begin{array}{c} \%\text{ Lysis }=\;[({\text{Signal}}_{\text{Test article}}–{\text{ Signal}}_{\text{untreated effector target cell control}}/\\ \left({\text{Signal}}_{\text{target cell maximum control}}–{\text{ Signal}}_{\text{target cells spontaneous control}}\right)]\\ \;\times \;100. \end{array}$$

To evaluate the effect of EGFR-TKIs on the TAVO412-mediated ADCC killing, NCI-H1975 and HCC827 cells were pre-treated with osimertinib or lazertinib for 48 h at 37°C before undergoing the ADCC assay as described above.

### ADCP assays

Phagocytosis was evaluated with human peripheral blood monocyte–derived macrophages as effector cells. Monocytes were isolated from previously frozen human PBMCs using EasySep™ Human Monocyte Enrichment Kit (StemCell) and were induced to differentiate into macrophages with macrophage colony stimulating factor (M-CSF) (StemCell) and interferon gamma (IFNγ) (StemCell) according to the manufacturer’s instructions. NSCLC target cells were labeled with Carboxy Fluorescein Succinimidyl Ester (CFSE) using the CFSE-Cell Labeling KIT (Abcam). Fifty thousand cancer cells per well were cocultured with 100,000 macrophages per well with the test articles for 24 h at 37°C. Next, Alexa-647-labeled anti-CD14 and anti-CD11b antibodies (R&D) were added to the culture and then incubated for 30-min at 4°C to label the macrophages. Flow cytometry (Beckman CytoFLEX) detected CSFE (FITC-A channel) positive cells and Alexa 647 (APC-A channel) positive cells. The cells initially were gated based on FSC vs SSC to eliminate debris and define a population gate (P1). P1 was then analyzed on FSC-H vs. FSC-A to isolate single cells (P2). A quadrant gate divided the P2 cells into four sub-populations with FITC-A vs APC-A (FITC^+^ APC^+^; FITC^-^ APC^+^; FITC^+^ APC^-^; FITC^-^ APC^-^) and the percentage of Q3 (FITC^+^ APC^-^) was calculated. CytExpert 2.4 software (Beckman Coulter) was used to calculate the killing percentage (%) as plotted on the y axis against the antibody concentration on the x axis using a four-parameter logistic (4PL) model. EC_50_ values, efficacy (y-axis span) and 95% CI for EC_50_ were calculated in GraphPad Prism 9.3.1(GraphPad Software, Inc.). Killing percentage (%) is determined by the following equation (14): % Killing = 100 x {(average %FITC^+^APC-A^-^ of [lowest mAb] for each antibody -%FITC^+^ APC-A^-^ sample)/(average %FITC^+^ APC-A^-^ of [lowest mAb] for each antibody)}.

### CDC assays

Twenty thousand tumor cells in RPMI 1640 medium were plated per well in 96-well plates. Upon the addition of test articles, the cells were incubated for 1 h at room temperature. After a 2-fold diluted Baby Rabbit Complement (Cedarlane) was added, the wells were incubated at 37°C for 1 h. The release of lactate dehydrogenase (LDH) in the supernatants was measured with the Roche Cytotoxicity Detection Kit. Control wells included: the target cells and complement at the lowest concentration of test antibody (TC spontaneous release); target cells only (T spontaneous release); and target cells plus 0.2% (w/v) Triton X-100 (maximum release). The lysis ratio on the y axis was plotted against the antibody concentration on the x axis using a four-parameter logistic (4PL) model. EC_50_ values, efficacy (y-axis span) and 95% CI for EC_50_ were calculated in GraphPad Prism 9.3.1(GraphPad Software, Inc.). The lysis ratio (%) was determined by the following equation with OD values (492 nm - 650 nm):


$$\begin{array}{c} \%\text{ Lysis }=\;[\left(\text{Experimental}-\text{TC spontaneous}\right)/(\text{Maximum release}\\ -\text{T spontaneous})]\;\times \;100\%. \end{array}$$

### *In vivo* efficacy studies in mice

All procedures related to animal care, handling, and treatment were carried out in accordance with the guidelines of GenePharma’s Institutional Animal Care and Use Committee (IACUC).

Tumor cells were injected subcutaneously into female Balb/c Nude mice (n = 5 or 7 per group; 6–10 weeks old; Nanjing GemPharmatech Co., Ltd.) at the right flank (5x10^6^ cells in 50% Matrigel admixed with 50% PBS for HCC827; 5x10^6^ NCI-H1975 in PBS; 1x10^7^ NCI-H292 in PBS). Therapeutic treatments began when the mean tumor volume reached approximately 200 mm^3^ and the first day of dosing was denoted as day 0. The detailed treatment regimens were described in each figure legend. In certain studies, at the end of the research, tumors were collected, weighed, and/or photographed. The average tumor volume in each group was calculated as length x width^2^ x 0.5 with units of mm^3^. Tumor volumes and body weights were recorded twice weekly, and the tumor growth curves of each treatment group were plotted as mean ± SEM. Tumor regression was defined as partial regression (PR) if the tumor volume decreased to 50% of the tumor volume at the start of treatment and as complete regression (CR) if the tumor volume too small to be recorded (tumor volume ~ 0 mm^3^).

### Statistical analysis of *in vivo* results

Statistical analysis was performed using GraphPad Prism software version 9.3.1 (GraphPad Software, Inc.). A comparison between two groups was performed using the Student’s T-test. Multiple group comparisons used a parametric one-way ANOVA followed by *post hoc* test (Tukey’s test). P values less than 0.05 were considered statistically significant.

## Data Availability

The original contributions presented in the study are included in the article/**Supplementary Material**. Further inquiries can be directed to the corresponding author.

## Glossary

ADCC: Antibody-Dependent Cellular Cytotoxicity
ADCP: Antibody-Dependent Cellular Phagocytosis
ADCT: Antibody-Dependent Cellular Trogocytosis
CDC: Complement-Dependent Cytotoxicity
CFSE: Carboxyfluorescein succinimidyl ester
cMET: Mesenchymal Epithelial Transition Factor
CI: Confidence Interval
CRT: Chemoradiotherapy
EGF: Epidermal Growth Factor
EGFR: Epidermal Growth Factor Receptor
ELISA: Enzyme-Linked Immunosorbent Assay
FACS: Fluorescence-Activated Cell Sorting
FcγR: Fc gamma Receptor
GADPH: GlycerAldehyde-3-Phosphate Dehydrogenase
GM-CSF: Granulocyte-Macrophage Colony-Stimulating Factor
HGF: Hepatocyte Growth Factor
HRP: Horseradish Peroxidase
IgG: Immunoglobulin G
LDH: Lactate Dehydrogenase
NSCLC: Non-Small Cell Lung Cancer
OS: Overall Survival
PBMC: Peripheral Blood Mononuclear Cell
PBS: Phosphate Buffer Saline
PFS: Progression-Free Survival
RPMI: Roswell Park Memorial Institute
SDS-PAGE: Sodium Dodecyl Sulfate–Polyacrylamide Gel Electrophoresis
SEC: Size Exclusion Chromatography
SEM: Standard Error of the Mean
SOC: Standard of Care
TKI: Tyrosine Kinase Inhibitor
TR-FRET: Time Resolved-Fluorescence Resonance Energy Transfer
VEGF: Vascular Endothelial Growth Factor
VEGFR2: Vascular Endothelial Growth Factor Receptor 2
PK: Pharmacokinetics
DSF: Differential Scanning Fluorometry
Tm: melting temperature

## References

[1] Araghi M Mannani R Heidarnejad maleki A Hamidi A Rostami S Safa SH. Recent advances in non-small cell lung cancer targeted therapy; an update review. Cancer Cell Int. (2023) 23. doi: 10.1186/s12935-023-02990-y PMC10416536 37568193

[2] Majeed U Manochakian R Zhao Y Lou Y. Targeted therapy in advanced non-small cell lung cancer: current advances and future trends. J Hematol Oncol. (2021) 14. doi: 10.1186/s13045-021-01121-2 PMC8264982 34238332

[3] Sholl LM Aisner DL Varella-Garcia M Berry LD Dias-Santagata D Wistuba II. Multi-institutional oncogenic driver mutation analysis in lung adenocarcinoma: the lung cancer mutation consortium experience. J Thoracic Oncology. (2015) 10:768–77. doi: 10.1097/jto.0000000000000516 PMC4410843 25738220

[4] Zhang Y-L Yuan J-Q Wang K-F Fu X-H Han X-R Threapleton D. The prevalence of EGFR mutation in patients with non-small cell lung cancer: a systematic review and meta-analysis. Oncotarget. (2016) 7(48):78985–93. doi: 10.18632/oncotarget.12587 27738317 PMC5346692

[5] Cho BC Simi A Sabari J Vijayaraghavan S Moores S Spira A. Amivantamab, an epidermal growth factor receptor (EGFR) and mesenchymal-epithelial transition factor (MET) bispecific antibody, designed to enable multiple mechanisms of action and broad clinical applications. Clin Lung Cancer. (2023) 24:89–97. doi: 10.1016/j.cllc.2022.11.004 36481319

[6] Li AR Chitale D Riely GJ Pao W Miller VA Zakowski MF. EGFR mutations in lung adenocarcinomas. J Mol Diagnostics. (2008) 10:242–8. doi: 10.2353/jmoldx.2008.070178 PMC2329789 18403609

[7] Passaro A Mok T Peters S Popat S Ahn M-J de Marinis F. Recent advances on the role of EGFR tyrosine kinase inhibitors in the management of NSCLC with uncommon, non exon 20 insertions, EGFR mutations. J Thoracic Oncology. (2021) 16:764–73. doi: 10.1016/j.jtho.2020.12.002 33333327

[8] Nan X Xie C Yu X Liu J. EGFR TKI as first-line treatment for patients with advanced EGFR mutation-positive non-small-cell lung cancer. Oncotarget. (2017) 8:75712–26. doi: 10.18632/oncotarget.20095 29088904 PMC5650459

[9] Johnson M Garassino MC Mok T Mitsudomi T. Treatment strategies and outcomes for patients with EGFR-mutant non-small cell lung cancer resistant to EGFR tyrosine kinase inhibitors: Focus on novel therapies. Lung Cancer. (2022) 170:41–51. doi: 10.1016/j.lungcan.2022.05.011 35714425

[10] He J Huang Z Han L Gong Y Xie C. Mechanisms and management of 3rd−generation EGFR−TKI resistance in advanced non−small cell lung cancer (Review). Int J Oncol. (2021) 59:90. doi: 10.3892/ijo.2021.5270 34558640 PMC8562388

[11] Piper-Vallillo AJ Sequist LV Piotrowska Z. Emerging treatment paradigms for EGFR-mutant lung cancers progressing on osimertinib: A review. J Clin Oncology. (2020) 38:2926–36. doi: 10.1200/jco.19.03123 32552277

[12] Krumbach R Schüler J Hofmann M Giesemann T Fiebig H-H Beckers T. Primary resistance to cetuximab in a panel of patient-derived tumor xenograft models: Activation of MET as one mechanism for drug resistance. Eur J Cancer. (2011) 47:1231–43. doi: 10.1016/j.ejca.2010.12.019 21273060

[13] Moores SL Chiu ML Bushey BS Chevalier K Luistro L Dorn K. A Novel Bispecific Antibody Targeting EGFR and cMet Is Effective against EGFR Inhibitor–Resistant Lung Tumors. Cancer Res. (2016) 76:3942–53. doi: 10.1158/0008-5472.Can-15-2833 27216193

[14] Grugan KD Dorn K Jarantow SW Bushey BS Pardinas JR Laquerre S. Fc-mediated activity of EGFR x c-Met bispecific antibody JNJ-61186372 enhanced killing of lung cancer cells. mAbs. (2016) 9:114–26. doi: 10.1080/19420862.2016.1249079 PMC5240640 27786612

[15] Park K Haura EB Leighl NB Mitchell P Shu CA Girard N. Amivantamab in EGFR exon 20 insertion–mutated non–small-cell lung cancer progressing on platinum chemotherapy: initial results from the CHRYSALIS phase I study. J Clin Oncology. (2021) 39:3391–402. doi: 10.1200/jco.21.00662 PMC8791812 34339292

[16] Guo MZ Marrone KA Spira A Freeman K Scott SC. Amivantamab: a potent novel EGFR/c-MET bispecific antibody therapy for EGFR-mutated non-small cell lung cancer. touchREVIEWS in Oncol & Haematol. (2021) 17(1):42–7. doi: 10.17925/OHR.2021.17.1.42

[17] Vyse S Huang PH. Amivantamab for the treatment ofEGFRexon 20 insertion mutant non-small cell lung cancer. Expert Rev Anticancer Ther. (2021) 22:3–16. doi: 10.1080/14737140.2022.2016397 34913823 PMC11614048

[18] Pacheco JM. Mobocertinib: A potential treatment for NSCLC with EGFR exon 20 insertions. Cancer Discovery. (2021) 11:1617–9. doi: 10.1158/2159-8290.Cd-21-0379 34284994

[19] Le X Nilsson M Goldman J Reck M Nakagawa K Kato T. Dual EGFR-VEGF pathway inhibition: A promising strategy for patients with EGFR-mutant NSCLC. J Thoracic Oncology. (2021) 16:205–15. doi: 10.1016/j.jtho.2020.10.006 33096270

[20] Nilsson MB Robichaux J Herynk MH Cascone T Le X Elamin Y. Altered regulation of HIF-1α in naive- and drug-resistant EGFR-mutant NSCLC: implications for a vascular endothelial growth factor-dependent phenotype. J Thoracic Oncology. (2021) 16:439–51. doi: 10.1016/j.jtho.2020.11.022 PMC8207565 33309987

[21] Lichtenberger BM Tan PK Niederleithner H Ferrara N Petzelbauer P Sibilia M. Autocrine VEGF signaling synergizes with EGFR in tumor cells to promote epithelial cancer development. Cell. (2010) 140:268–79. doi: 10.1016/j.cell.2009.12.046 20141840

[22] Cohen MH Gootenberg J Keegan P Pazdur R. FDA drug approval summary: bevacizumab (Avastin^®^) plus carboplatin and paclitaxel as first-line treatment of advanced/metastatic recurrent nonsquamous non-small cell lung cancer. Oncologist. (2007) 12:713–8. doi: 10.1634/theoncologist.12-6-713 17602060

[23] Larkins E Scepura B Blumenthal GM Bloomquist E Tang S Biable M. Food and drug administration approval summary: ramucirumab for the treatment of metastatic non-small cell lung cancer following disease progression on or after platinum-based chemotherapy. Oncologist. (2015) 20:1320–5. doi: 10.1634/theoncologist.2015-0221 PMC4718430 26446239

[24] Masuda C Yanagisawa M Yorozu K Kurasawa M Furugaki K Ishikura N. Bevacizumab counteracts VEGF-dependent resistance to erlotinib in an EGFR-mutated NSCLC xenograft model. Int J Oncology. (2017) 51:425–34. doi: 10.3892/ijo.2017.4036 PMC5504975 28627678

[25] Raghav KP Gonzalez-Angulo AM Blumenschein GR Jr. Role of HGF/MET axis in resistance of lung cancer to contemporary management. Transl Lung Cancer Res. (2012) 1:179–93. doi: 10.3978/j.issn.2218-6751.2012.09.04 PMC4367559 25806180

[26] Takeuchi S Wang W Li Q Yamada T Kita K Donev IS. Dual inhibition of met kinase and angiogenesis to overcome HGF-induced EGFR-TKI resistance in EGFR mutant lung cancer. Am J Pathology. (2012) 181:1034–43. doi: 10.1016/j.ajpath.2012.05.023 22789825

[27] Rivera-Soto R Henley B Pulgar MA Lehman SL Gupta H Perez-Vale KZ. Amivantamab efficacy in wild-type EGFR NSCLC tumors correlates with levels of ligand expression. NPJ Precis Oncol. (2024) 8:192. doi: 10.1038/s41698-024-00682-y 39242834 PMC11379809

[28] Liu R Oldham R Teal E Beers S Cragg M. Fc-engineering for modulated effector functions—Improving antibodies for cancer treatment. Antibodies. (2020) 9. doi: 10.3390/antib9040064 PMC7709126 33212886

[29] Stavenhagen JB Gorlatov S Tuaillon N Rankin CT Li H Burke S. Fc Optimization of Therapeutic Antibodies Enhances Their Ability to Kill Tumor Cells*In vitro*and Controls Tumor Expansion*In vivo*via Low-Affinity Activating Fcγ Receptors. Cancer Res. (2007) 67:8882–90. doi: 10.1158/0008-5472.Can-07-0696 17875730

[30] Varsano S Rashkovsky L Shapiro H Ophir D Mark-Bentankur T. Human lung cancer cell lines express cell membrane complement inhibitory proteins and are extremely resistant to complement-mediated lysis; a comparison with normal human respiratory epithelium *in vitro*, and an insight into mechanism(s) of resistance. Clin Exp Immunol. (1998) 113:173–82. doi: 10.1046/j.1365-2249.1998.00581.x PMC1905035 9717965

[31] Shu CA Goto K Ohe Y Besse B Lee S-H Wang Y. Amivantamab and lazertinib in patients with EGFR-mutant non–small cell lung (NSCLC) after progression on osimertinib and platinum-based chemotherapy: Updated results from CHRYSALIS-2. J Clin Oncol. (2022) 40:9006–6. doi: 10.1200/JCO.2022.40.16_suppl.9006 39755170

[32] Teng K Zhang Y Hu X Ding Y Gong R Liu L. Nimotuzumab enhances radiation sensitivity of NSCLC H292 cells *in vitro* by blocking epidermal growth factor receptor nuclear translocation and inhibiting radiation-induced DNA damage repair. Onco Targets Ther. (2015) 8:809–18. doi: 10.2147/ott.S77283 PMC4403694 25926742

[33] Nakade J Takeuchi S Nakagawa T Ishikawa D Sano T Nanjo S. Triple inhibition of EGFR, met, and VEGF suppresses regrowth of HGF-triggered, erlotinib-resistant lung cancer harboring an EGFR mutation. J Thoracic Oncology. (2014) 9:775–83. doi: 10.1097/jto.0000000000000170 PMC4132034 24828661

[34] Vijayaraghavan S Lipfert L Chevalier K Bushey BS Henley B Lenhart R. Amivantamab (JNJ-61186372), an fc enhanced EGFR/cMet bispecific antibody, induces receptor downmodulation and antitumor activity by monocyte/macrophage trogocytosis. Mol Cancer Ther. (2020) 19:2044–56. doi: 10.1158/1535-7163.Mct-20-0071 32747419

[35] Weisser NE Sanches M Escobar-Cabrera E O’Toole J Whalen E Chan PWY. An anti-HER2 biparatopic antibody that induces unique HER2 clustering and complement-dependent cytotoxicity. Nat Commun. (2023) 14:1394. doi: 10.1038/s41467-023-37029-3 36914633 PMC10011572

[36] Chen J Liao S Xiao Z Pan Q Wang X Shen K. The development and improvement of immunodeficient mice and humanized immune system mouse models. Front Immunol. (2022) 13:1007579. doi: 10.3389/fimmu.2022.1007579 36341323 PMC9626807

[37] Jin Y Chen P Zhou H Mu G Wu S Zha Z. Developing transcriptomic biomarkers for TAVO412 utilizing next generation sequencing analyses of preclinical tumor models [Original Research. Front Immunol. (2025) 16:1505868. doi: 10.3389/fimmu.2025.1505868 39995668 PMC11847686

[38] Steiner P Joynes C Bassi R Wang S Tonra JR Hadari YR. Tumor growth inhibition with cetuximab and chemotherapy in non–small cell lung cancer xenografts expressing wild-type and mutated epidermal growth factor receptor. Clin Cancer Res. (2007) 13:1540–51. doi: 10.1158/1078-0432.Ccr-06-1887 17332300

[39] Tang Y Lou J Alpaugh RK Robinson MK Marks JD Weiner LM. Regulation of antibody-dependent cellular cytotoxicity by igG intrinsic and apparent affinity for target antigen. J Immunol. (2007) 179:2815–23. doi: 10.4049/jimmunol.179.5.2815 17709495

[40] Cavazzoni A Alfieri RR Cretella D Saccani F Ampollini L Galetti M. Combined use of anti-ErbB monoclonal antibodies and erlotinib enhances antibody-dependent cellular cytotoxicity of wild-type erlotinib-sensitive NSCLC cell lines. Mol Cancer. (2012) 11:91. doi: 10.1186/1476-4598-11-91 23234355 PMC3577499

[41] BjÖRkelund H Gedda L Malmqvist M Andersson K. Resolving the EGF-EGFR interaction characteristics through a multiple-temperature, multiple-inhibitor, real-time interaction analysis approach. Mol Clin Oncology. (2013) 1:343–52. doi: 10.3892/mco.2012.37 PMC3956273 24649173

[42] Buday L Björkelund H Gedda L Barta P Malmqvist M Andersson K. Gefitinib induces epidermal growth factor receptor dimers which alters the interaction characteristics with 125I-EGF. PloS One. (2011) 6. doi: 10.1371/journal.pone.0024739 PMC3171474 21931838

[43] Arteaga CL Ramsey TT Shawver LK Guyer CA. Unliganded epidermal growth factor receptor dimerization induced by direct interaction of quinazolines with the ATP binding site. J Biol Chem. (1997) 272:23247–54. doi: 10.1074/jbc.272.37.23247 9287333

[44] Gan HK Walker F Burgess AW Rigopoulos A Scott AM Johns TG. The epidermal growth factor receptor (EGFR) tyrosine kinase inhibitor AG1478 increases the formation of inactive untethered EGFR dimers. J Biol Chem. (2007) 282:2840–50. doi: 10.1074/jbc.M605136200 17092939

[45] Kim H Kim SH Kim MJ Kim SJ Park SJ Chung JS. EGFR inhibitors enhanced the susceptibility to NK cell-mediated lysis of lung cancer cells. J Immunother. (2011) 34:372–81. doi: 10.1097/CJI.0b013e31821b724a 21499124

[46] Upadhya A Yadav KS Misra A. Targeted drug therapy in non-small cell lung cancer: Clinical significance and possible solutions-Part I. Expert Opin Drug Deliv. (2021) 18:73–102. doi: 10.1080/17425247.2021.1825377 32954834

[47] Wrona A Dziadziuszko R Jassem J. Combining radiotherapy with targeted therapies in non-small cell lung cancer: focus on anti-EGFR, anti-ALK and anti-angiogenic agents. Trans Lung Cancer Res. (2021) 10:2032–47. doi: 10.21037/tlcr-20-552 PMC8107745 34012812

[48] Raben D Helfrich B Chan DC Ciardiello F Zhao L Franklin W. The effects of cetuximab alone and in combination with radiation and/or chemotherapy in lung cancer. Clin Cancer Res. (2005) 11:795–805. doi: 10.1158/1078-0432.795.11.2 15701870

[49] De Bacco F Luraghi P Medico E Reato G Girolami F Perera T. Induction of MET by ionizing radiation and its role in radioresistance and invasive growth of cancer. JNCI: J Natl Cancer Institute. (2011) 103:645–61. doi: 10.1093/jnci/djr093 21464397

[50] Gao H Xue J Zhou L Lan J He J Na F. Bevacizumab radiosensitizes non-small cell lung cancer xenografts by inhibiting DNA double-strand break repair in endothelial cells. Cancer Letters. (2015) 365:79–88. doi: 10.1016/j.canlet.2015.05.011 25982206

[51] Yang Z Tam KY. Combination strategies using EGFR-TKi in NSCLC therapy: learning from the gap between pre-clinical results and clinical outcomes. Int J Biol Sci. (2018) 14:204–16. doi: 10.7150/ijbs.22955 PMC5821041 29483838

[52] Labrijn AF Meesters JI Priem P de Jong RN van den Bremer ETJ van Kampen MD. Controlled Fab-arm exchange for the generation of stable bispecific IgG1. Nat Protoc. (2014) 9:2450–63. doi: 10.1038/nprot.2014.169 25255089

