# Supplementary material for: A trispecific antibody targeting EGFR/cMET/VEGF-A demonstrates multiple mechanisms of action to inhibit wild-type and mutant NSCLC animal models
Source: Front Oncol. 2025 May 16;15:1533059. doi: 10.3389/fonc.2025.1533059 (PMC12122299; doi:10.3389/fonc.2025.1533059)
Supplement: Supplementary file 1 [file DataSheet1.docx]

**A Trispecific Antibody targeting EGFR/cMET/VEGF-A Demonstrates Multiple Mechanisms of Action to inhibit Wild-Type and Mutant NSCLC Animal Models**

**Authors: Ying Jin^1^, Ping Sun^1^, Peng Chen^1^, Yuqiang Xu^1^, Guangmao Mu^1^, Zhengxia Zha^1^, Simin Wu^1^, Meixia Fu^1^, Hao Jiang^1^, Sheng Huang^1^, Fulai Zhou^1^, Chao Han ^1, 2^, Mark L. Chiu^1, 2^**

1. Research & Development Department, Tavotek Biotherapeutics, Suzhou, Jiang Su, China.

2. Research & Development, Tavotek Biotherapeutics, Spring House, Pennsylvania, USA.

**Correspondence:**

**Mark L Chiu, PhD**

Research and Discovery Department

Tavotek Biotherapeutics

Building B, Phase 1, Life Bay, WuZhong Biomedical Industry Park, 999 Yinshan Lake Road, Wuzhong District, Suzhou City

727 Norristown Road, 3 Spring House Innovation Park Suite 101, Lower Gwynedd, Pennsylvania 19002

**Email:** [Mark.Chiu@tavotek.com](mailto:Mark.Chiu@tavotek.com)

Supplementary Methods and Materials

ELISA analysis of VEGF level in cell supernatants

Four thousand cells per well were seeded in a 96-well plate with complete medium. After 24 h of incubation, the cells were harvested by centrifugation and counted. The supernatants of the conditioned media were frozen until analysis. The concentrations of VEGF in the media were measured using the VEGF ELISA kit (BioLegend, #446504) according to the manufacturer's instructions. The VEGF level in each sample was then normalized to a cell number of 100,000.

EGFR/cMET receptor density measurements

Densities of EGFR and cMET on tumor cell lines were determined by flow cytometry. Briefly, the phycoerythrin (PE)-labelled polyclonal antibodies- anti-cMET antibody (Sinobiological, #10692-R243-P) and anti-human EGFR antibody (BioLegend, #352904)- were used as primary antibodies for detecting surface EGFR and cMET on cell lines. The gMFI levels were determined by flow cytometry (described in Materials and Methods). The receptor densities were quantified by comparison to a standard curve generated by QuantiBRITE^TM^ PE beads (BD Biosciences, 340495)(47).

To assess the impact of EGFR-TKIs on the EGFR/cMET receptor density, NCI-H1975 and HCC827 cells were co-cultured with specified dilutions of osimertinib and lazertinib, respectively, for 48 h at 37℃. The gMFI levels EGFR and cMET receptor were then measured as described above.

Surrogate ADCC and ADCP reporter assays

The ADCC and ADCP surrogate reporter assays were performed using the Bio-Lite^TM^ Luciferase Assay System (Vazyme, DD1201-02). The target cells were harvested and plated at 20,000 cells per well in RPMI1640 (Gibco, 61870036). Serial dilutions of test antibodies were added, followed by a 15 min incubation at 37°C. Next, the reporter cells [ADCC: Jurkat-FcγRIIIa V158 cells (Vazyme, DD1301); ADCP: Jurkat-FcγRIIa-H131(Vazyme, DD1304-01)] were added at 200,000 cells per well, and incubation was continued for 6 h at 37°C. Plates were equilibrated at room temperature for 15 min. Then Bio-Lite reagent was added to each well and luminescence was detected using the Tecan Spark microplate reader. Antibody concentration vs. relative luminescence units (RLUs) were plotted as a “best fit” curve with GraphPad Prism version 9.3.1 using a Variable Slope (log(agonist); Hill Slope; 4 parameters). Nonlinear regression was used for the analysis. EC50 values, efficacy (y-axis span) and 95% CI for EC50 were calculated in GraphPad Prism 9.3.1(GraphPad Software, Inc.).

*In vivo* efficacy studies in mice

Mouse *in vivo* efficacy studies were carried out as described in the Material and Methods section, except that 5 to 6 mice were used per group and the mean tumor volume to start treatment ranged from 100 to 300 mm^3^.

To conduct a stand-alone pharmacodynamic study evaluating the total levels of EGFR and cMET receptor expression in tumors after TAVO412 treatment, tumor-bearing mice were treated with either an isotype control or TAVO412 for two doses with a 2-day interval via intraperitoneal (IP) injection. Then the tumors were resected 24 h after the second dose and snap-frozen in liquid nitrogen for western blot analysis.

*In vivo* PK study

A PK study was conducted in non-tumor-bearing female Balb/c nude mice (*n* = 3) of the same strain as used in the aforementioned *in vivo* efficacy study. TAVO412 was administered intraperitoneally at doses of 1 and 3 mg/kg, and serum samples were collected for up to 14 d (336 h) post-injection. Serum concentrations of TAVO412 were quantified using a commercially available Human IgG Precoated ELISA Kit (Dakewe; Cat. #1128162). The half-life was estimated using the noncompartmental analysis module in WinNonlin® (version 8.3; Certara, Mountain View, CA, USA).

The procedures related to animal care, handling, and treatment were carried out in accordance with the guidelines of GenePharma's Institutional Animal Care and Use Committee (IACUC).

Western blotting

Frozen tumor samples were lysed in ice-cold RIPA buffer (Thermo Fisher, #89900) containing protease inhibitor cocktail (Roche, #11697498001) and phosphatase inhibitor cocktail (Roche, #04906837001) using a homogenizer (Shanghai Jingxin Experimental Technology). The lysates were then cleared by centrifugation, and the protein concentrations were determined using the BCA Protein Assay. The samples were heated at 95°C for 10 min prior to electrophoresis. The protein extracts were separated by electrophoresis on precast Sure PAGE 4–12% gradient Bis-Tris gels from GenScript (#M00654) and transferred to PVDF membranes (IPVH00010, Millipore) using electroblotting. The membranes were then probed with primary antibodies including anti-EGFR (Santa Cruz Biotechnology, sc-373746), anti-cMET (Santa Cruz Biotechnology, sc-514148), or anti-GAPDH (Cell Signaling Technology, 3683S). A chemiluminescence detection kit (Beijing Lablead Biotech, E1060) was used to visualize the protein bands, and the e-BLOT WB IMAGER (China, Shanghai) was employed to acquire the image. Band intensities were analyzed using ImageJ software, and the average total protein relative to the loading control (GAPDH) was graphed.

Supplementary Figures


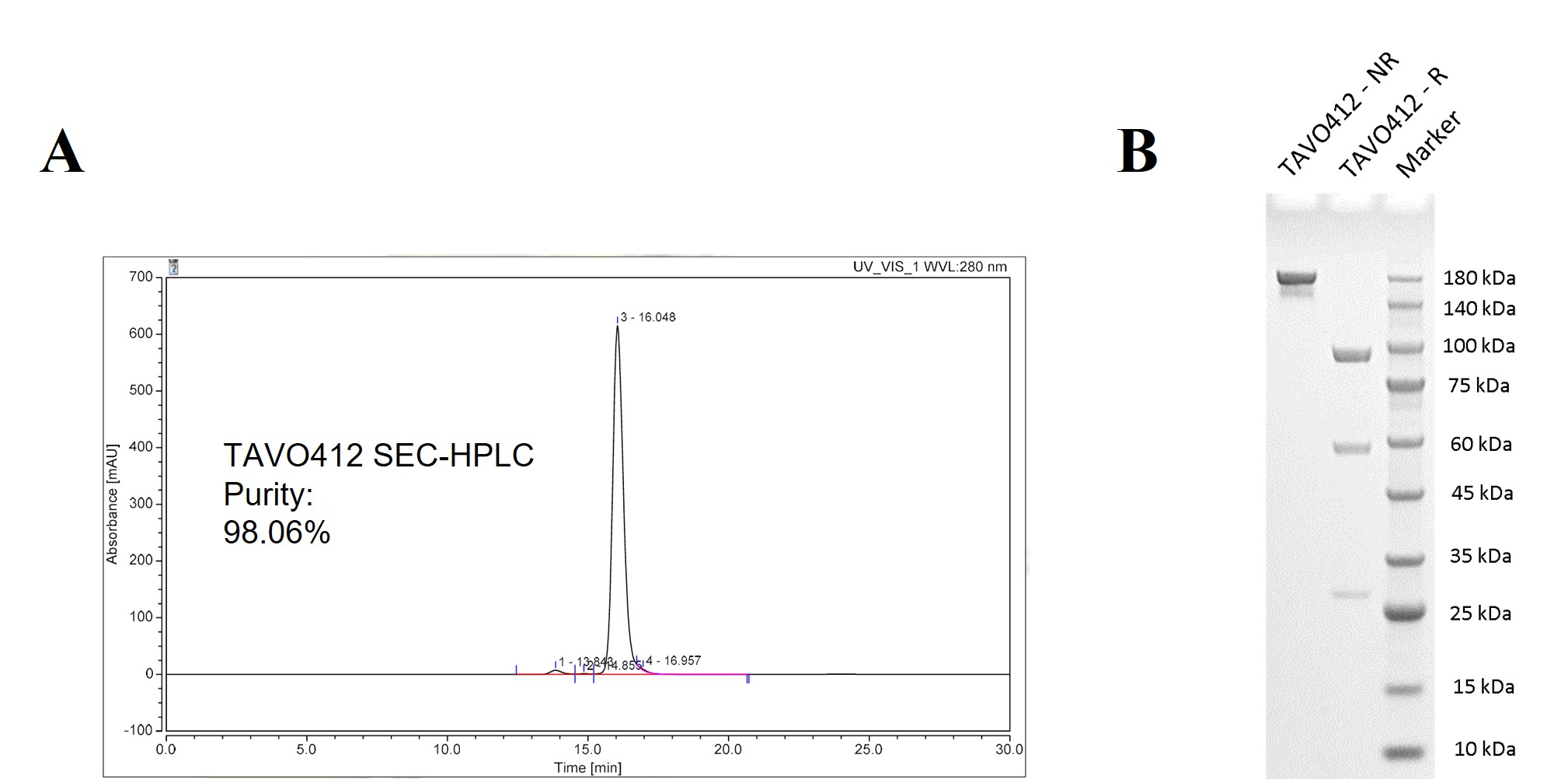


**Supplementary Figure S1. Characterization of TAVO412 purity and molecular integrity**

(**A**) SEC-HPLC chromatogram showing monomeric purity of TAVO412 after purification (98.06%). (**B**) SDS-PAGE under non-reducing (NR) and reducing (R) conditions confirmed proper assembly. Expected size: ~75~100 kDa HC1, 45~60 kDa HC2 and 25~35 kDa LC upon Coomassie staining. Molecular weight markers are shown in the right lane. The abbreviations were: SEC-HPLC, size-exclusion chromatography-high performance liquid chromatography; SDS-PAGE, sodium dodecyl sulfate–polyacrylamide gel electrophoresis; NR, non-reducing condition; R, reducing condition; HC1, heavy chain 1; HC2, heavy chain 2; LC, light chain; kDa: kilodalton


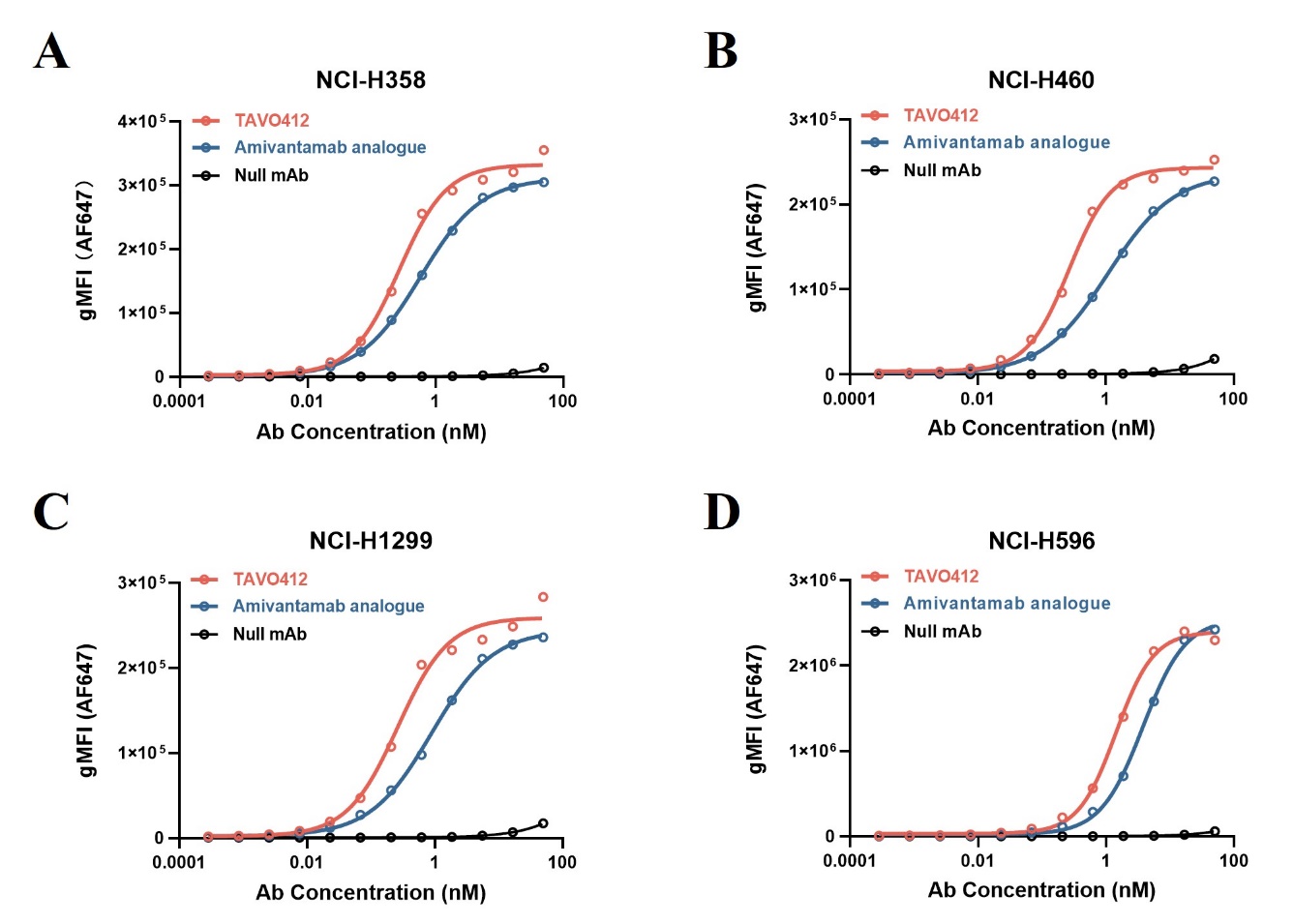


**Supplementary Figure S2****. Selected NSCLC cell binding of TAVO412 and amivantamab analogue were comparable**. Binding of TAVO412 (red open circle), Amivantamab analogue (blue open circle), and null mAb (black open circle) to (**A**) NCI-H358, (**B**) NCI-H460, (**C**) NCI-H1299 and (**D**) NCI-H596 cell lines was analyzed by flow cytometry. Reference to Supplementary Table S2 for corresponding EC50, 95% CI for EC50, and efficacy (span in y axis) values. The abbreviations were: CI, confidence interval; EC50, half-maximal effective concentration; nM, nanomolar; Ab, antibody; gMFI, geometric mean fluorescent intensity; AF647, Alexa Fluor 647 dye.


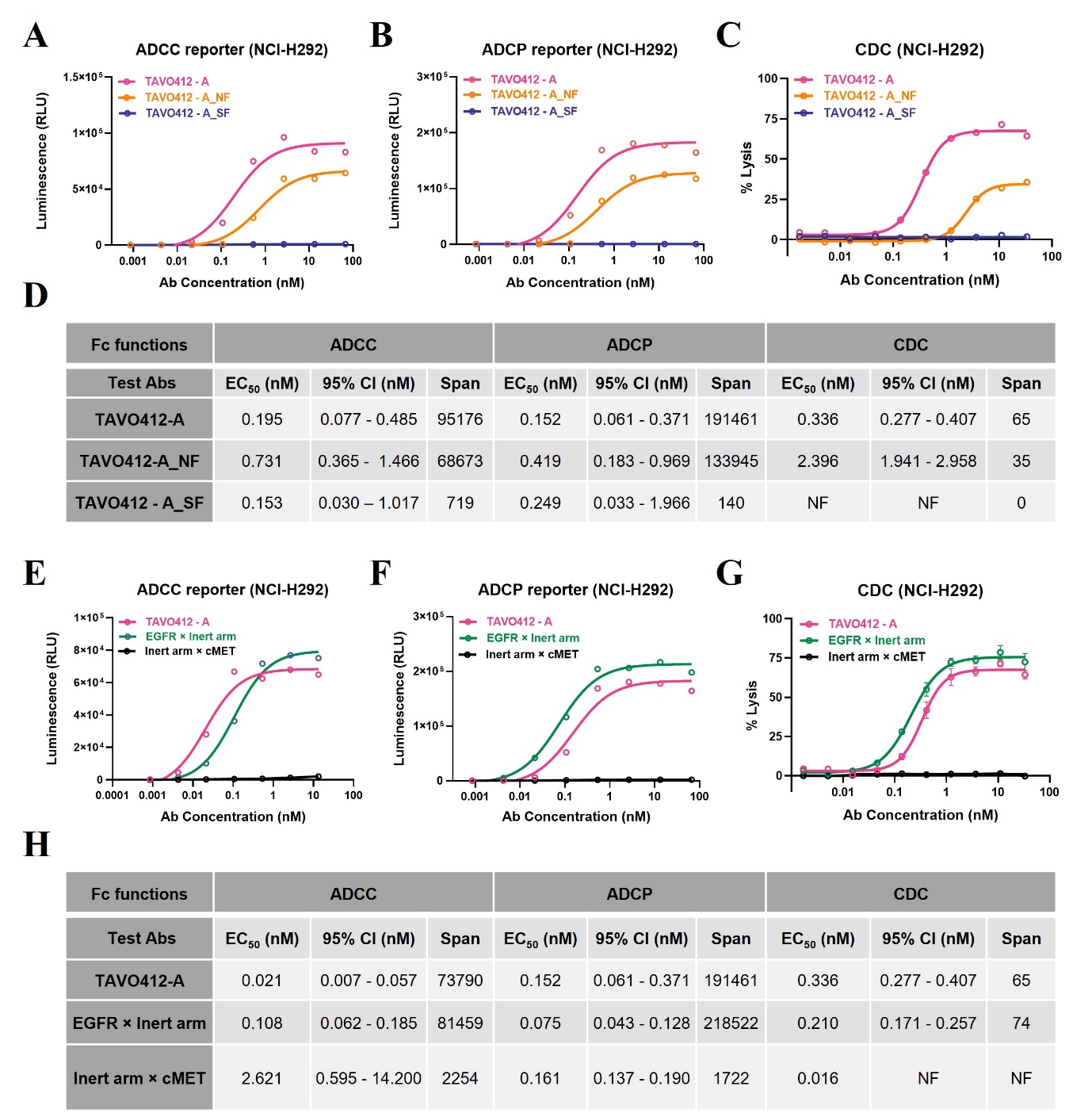


**Supplementary Figure S3****. Dual epitope EGFR arm mediates TAVO412-enhanced Fc effector functions** The Fc effector functions and target arm contributions were evaluated in NCI-H292**. (A-D)** Evaluation of Fc mutations. TAVO412-A (variant of TAVO412 without VEGF arm; magenta open circle), TAVO412-A_NF (TAVO412-A with normal Fc; orange open circle), TAVO412-A_SF (TAVO412-A with silenced Fc; purple open circle) and a null mAb control were tested for (**A**) ADCC (reporter assay), (**B**) ADCP (reporter assay) and (**C**) CDC (complement cell lysis assay). (**D**) The EC50, 95% CI for EC50 values and efficacy (y axes span) are in the table. (**E-H)** Binding arm contribution to Fc effector functions. TAVO412-A (magenta open circle), EGFR x Inert arm (green open circle) and Inert arm x cMET (black open circle) were compared for the activity of (**E**) ADCC (reporter assay), (**F**) ADCP (reporter assay) and (**G**) CDC. (**H**) The EC50, 95% CI for EC50 values and efficacy (y axis span) were summarized in the table. Representative data from 2 to 3 independent experiments are shown. The abbreviations were: CI, confidence interval; EC50, half-maximal effective concentration; nM, nanomolar; NF, no fit returned by GraphPad Prism; Ab, antibody.


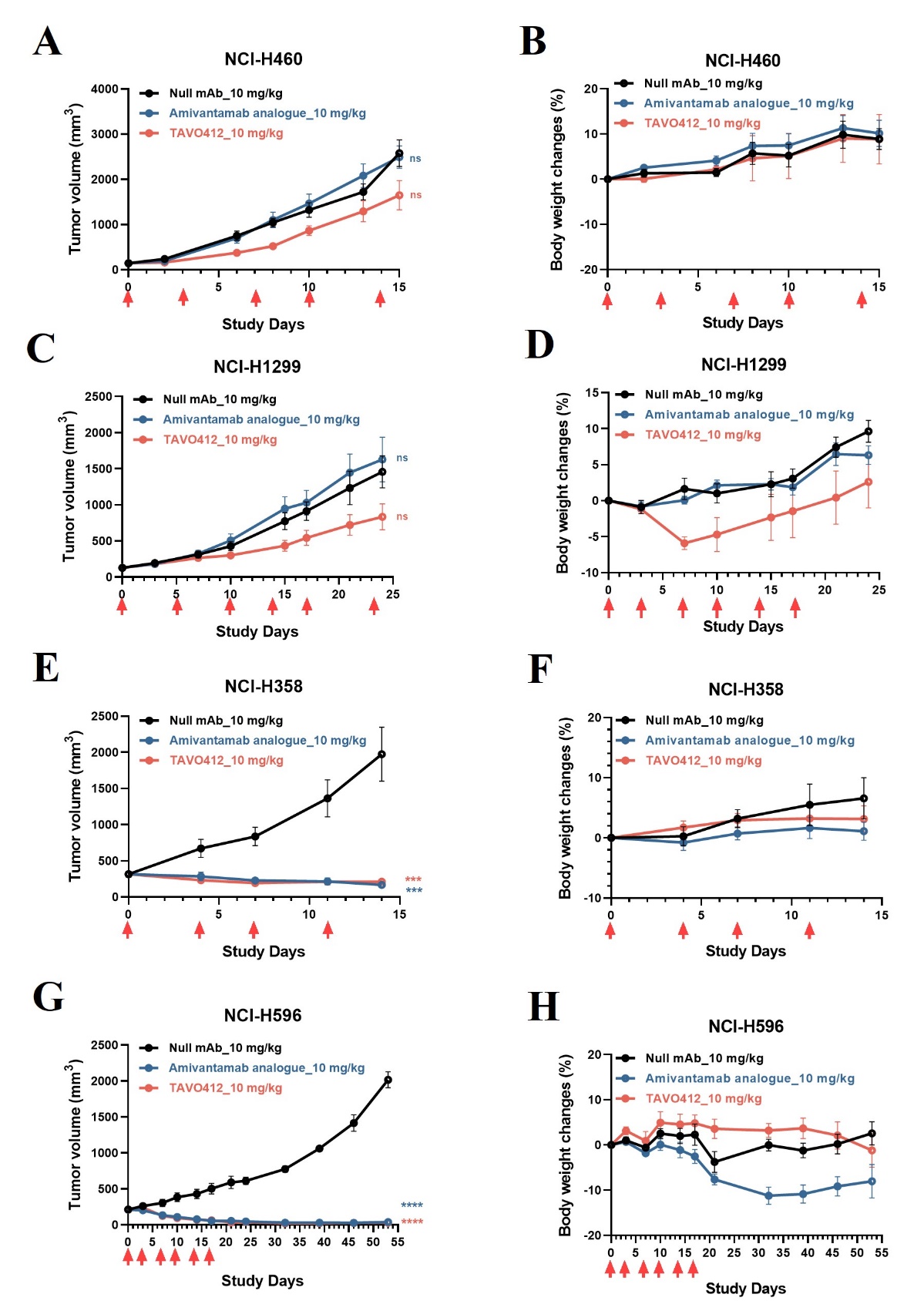


**Supplementary Figure S4. TAVO412 showed antitumor activity in NSCLC xenografts with varying receptor densities.** Antitumor activity was evaluated in a panel of four NSCLC xenograft models treated with TAVO412 (10 mg/kg; red open circle), Amivantamab analogue (10 mg/kg; blue open circle), and Null mAb (10 mg/kg; black open circle) antibodies. Tumor growth was monitored twice weekly for (**A**) NCI-H460, (**C**) NCI-H1299, (**E**) NCI-H358, and (**G**) NCI-H596. The body weight changes were monitored twice weekly for (**B**) NCI-H460, (**D**) NCI-H1299, (**F**) NCI-H358, and (**H**) NCI-H596. Red arrows indicate the dosing days. Data represent the mean ± SEM (n = 5, 6/group). **, P < 0.01; ns: not significant compared to control group. Statistical significance was calculated by one-way ANOVA followed by Tukey’s multiple comparisons test to compare each treatment group with the null mAb group. The abbreviations were: SEM, standard error of the mean.


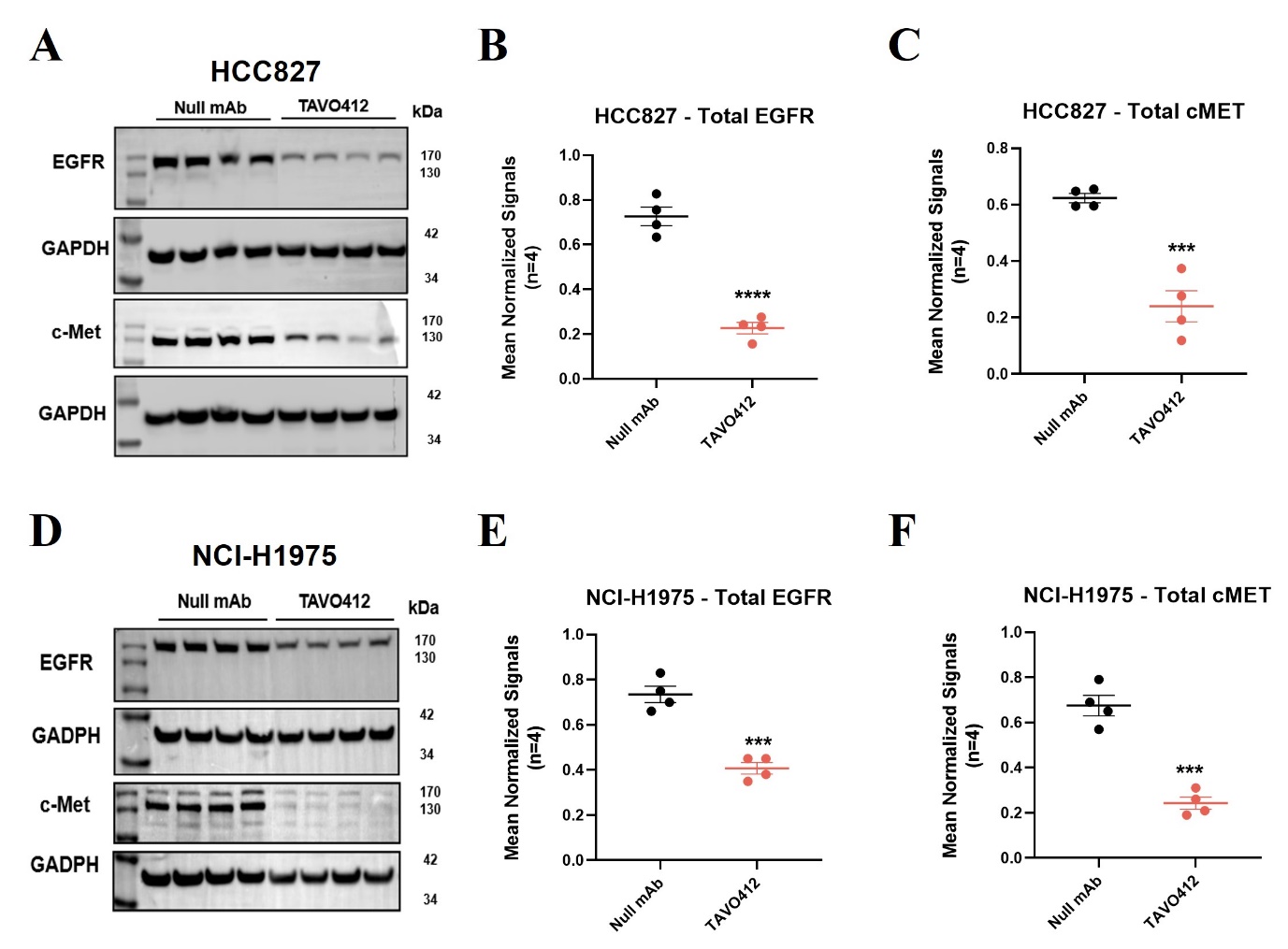


**Supplementary Figure S5. TAVO412 induced EGFR and cMET down-regulation in HCC827 and NCI-H1975 tumors.** Mice bearing the HCC827 (**A, B, C**) or NCI-H1975 (**D, E, F**) tumors were administered two doses of TAVO412 or null mAb at 10 mg/kg. (**A, D**) Tumors were collected 24 h after the 2^nd^ dose and analyzed by western blot for the protein markers shown to the left of the images. The treatment information is shown above of the lanes. Densitometry measurements for EGFR (**B, E**) and cMET (**C, F**) were normalized to loading control (GAPDH) and quantified as mean ± SEM in the scatter diagram (n = 4/group). The antibodies were labeled (B, C, E, and F): TAVO412: red closed circle; Null mAb (black closed circle). P values were calculated using Student's t test. ***, P <0.001; ****, P < 0.0001. The abbreviations were: kDa, kilodalton; GADPH, Glyceraldehyde-3-phosphate dehydrogenase; EGFR, epidermal growth factor receptor; c-Met, mesenchymal epithelial transition factor; SEM, standard error of the mean.


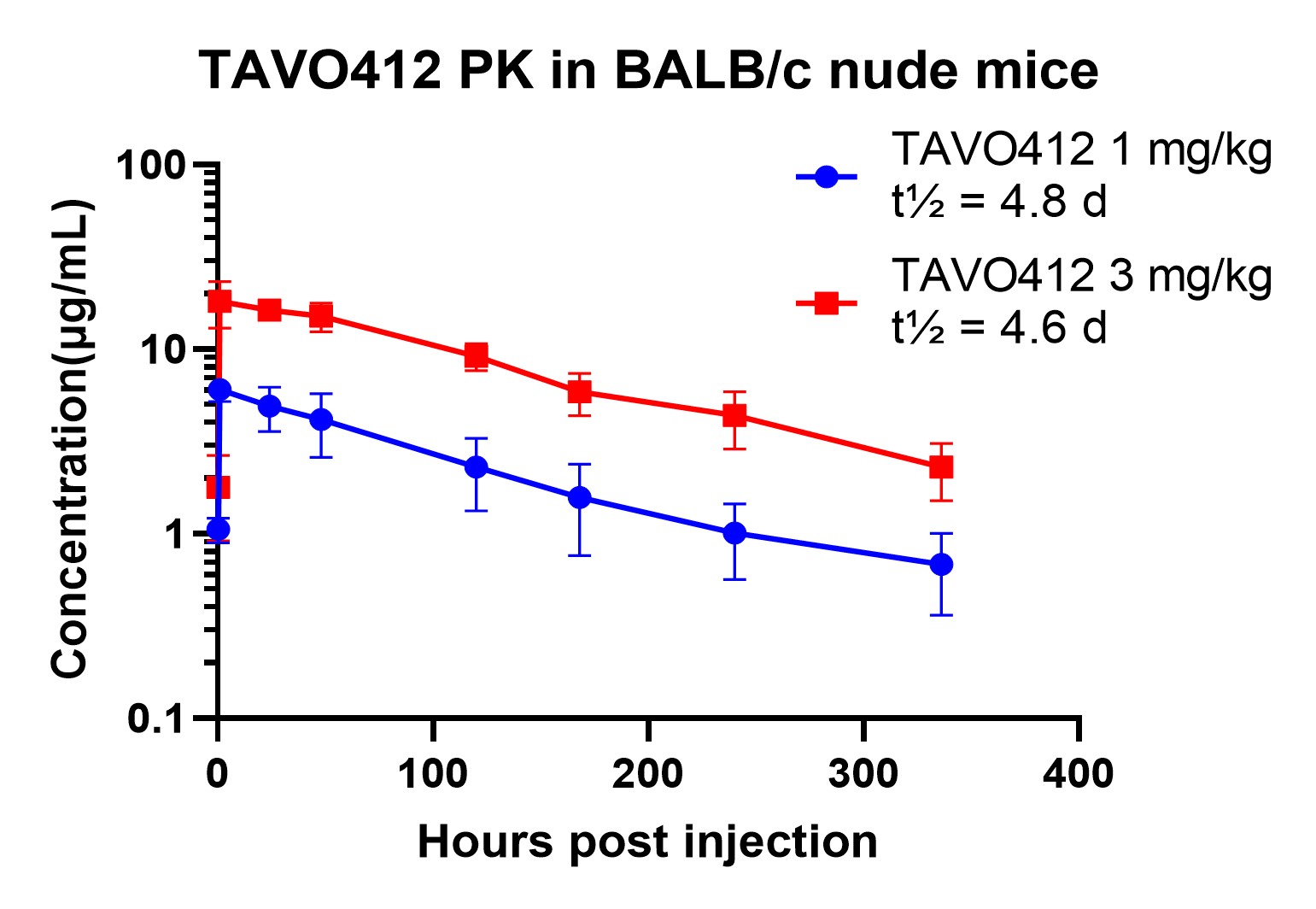


**Supplementary Figure S6. Pharmacokinetic profile of TAOV412 in Balb/c nude mice**

Serum concentration-time curve of TAVO412 following a single intraperitoneal (IP) administration at 1 and 3 mg/kg to female Balb/c nude mice (n=3/group). The elimination half-life (t½) was calculated by non-compartmental analysis in WinNonlin software. Data are presented as mean ± SEM. The abbreviations were: SEM, standard error of the mean.


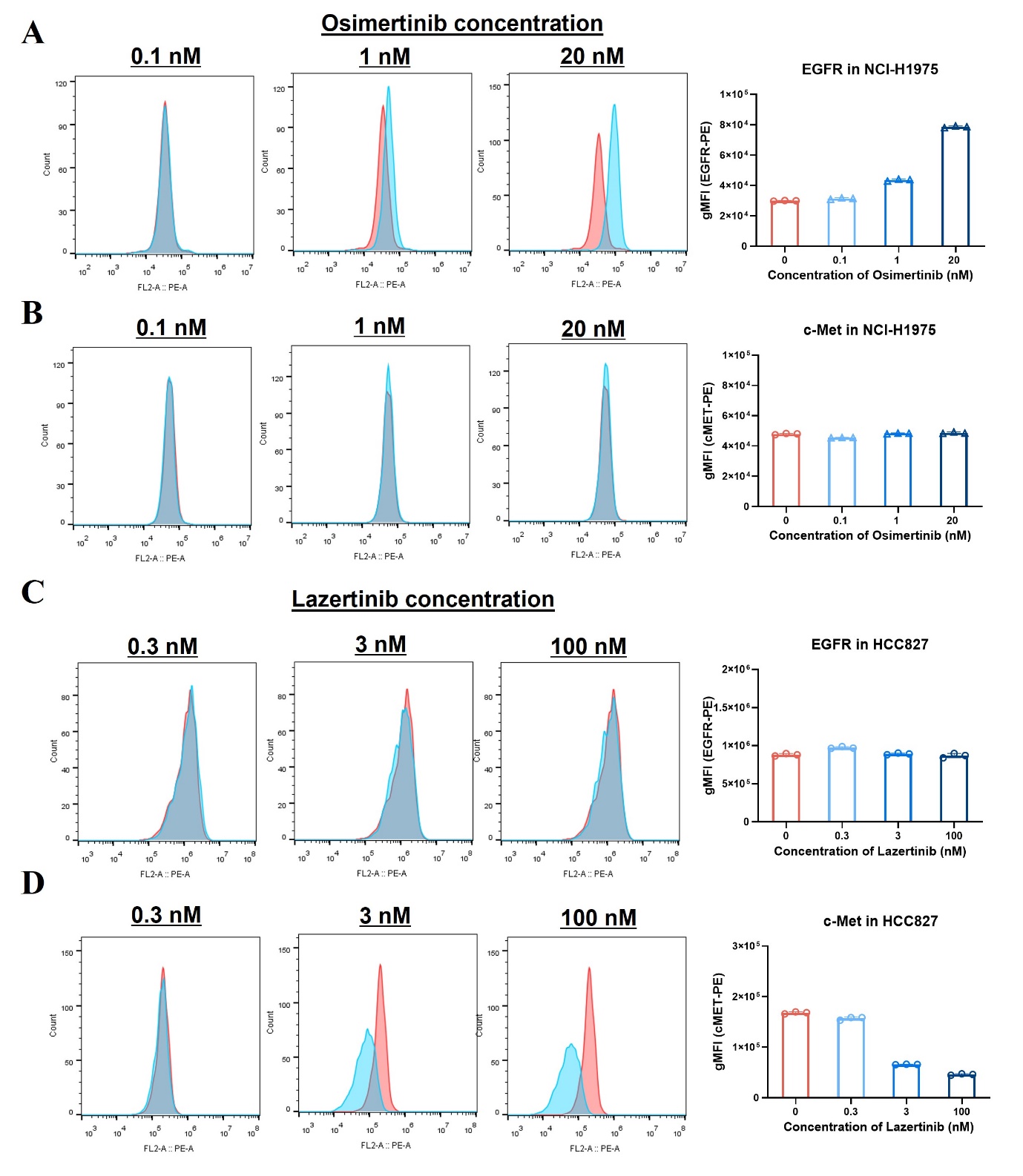


**Supplementary Figure S7****. EGFR-TKIs affected EGFR or cMET receptor densities in NCI-H1975 and HCC827 cells.** NCI-H1975 cells were treated with osimertinib at designated concentrations for 48 h. The cells were then assessed for changes in cell surface density of (**A**) EGFR and (**B**) cMET by flow cytometry. HCC827 cells were treated with lazertinib at designated concentrations for 48 h. The cells were then assessed for the alterations in the cell surface density by flow cytometry of (**C**) EGFR and (**D**) cMET. The flow cytometry histogram and the corresponding values of gMFI were displayed to show the alteration in gMFI intensity following treatment. The addition of osimertinib at concentration of 0, 0.1,1 and 20nM were labeled as red open circle, light, medium and dark blue open up triangle, respectively; The addition of Lazertinib at concentration of 0, 0.3,3 and 100 nM were labeled as red open circle, light, medium and dark blue open circle, respectively; The abbreviations were: gMFI, geometric mean fluorescent intensity; nM, nanomolar; TKI, tyrosine kinase inhibitors.

Supplementary Tables

**Supplementary Table S1: NSCLC Cell Line Characterization**

| **Cell lines** | **Genotype of EGFR** | **Genotype of cMET** | **Genotype of KRAS** | **Receptor density of EGFR** | **Receptor density of**  **c-Met** | **VEGF secretion level**  **(pg / mL)** |
| --- | --- | --- | --- | --- | --- | --- |
| **HCC827** | Exon19 Del; AMP | WT | WT | 1,505,000 | 104,000 | 1,700 |
| **NCI-H596** | WT | WT | WT | 177,000 | 7,500 | 3,500 |
| **NCI-H292** | WT | WT | WT | 137,000 | 17,000 | 1,200 |
| **NCI-H358** | WT | WT | G12C mutant | 33,000 | 25,000 | 5,600 |
| **NCI-H1975** | Exon21 L858R; T790M | WT | WT | 29,000 | 22,000 | 580 |
| **NCI-H1299** | WT | WT | WT | 19,300 | 7,600 | 370 |
| **NCI-H460** | WT | WT | Q61H mutant | 12,200 | 8,200 | 880 |

The genotype designations of the listed cell lines were from Cancer Cell Line Encyclopedia (https://depmap.org/portal). Levels of EGFR and cMET on the indicated tumor cell lines were determined by flow cytometry (Supplementary Methods). PE labelled anti-cMET polyclonal antibody and anti-human EGFR polyclonal antibody were used as primary antibodies for detecting surface EGFR and cMET on cell lines. The receptor density was quantified with a standard curve generated by QuantiBRITE^TM^ PE beads; Human VEGF protein in culture medium was assessed by ELISA as described in Supplementary Methods. The abbreviations were: WT, wild type; Del, deletion; AMP, amplification; pg, picogram; mL, milliliter

**Supplementary Table S2**
**Binding Avidity for Selected NSCLC Cell Lines**

| **Cell lines** | **Test Abs** | **EC50 (nM)** | **95% CI (nM)** | **Span** |
| --- | --- | --- | --- | --- |
| **NCI-H292** | **TAVO412** | 0.399 | NF | 520240 |
|  | **Amivantamab analogue** | 1.150 | NF | 517366 |
| **HCC827** | **TAVO412** | 1.037 | NF | 1349922 |
|  | **Amivantamab analogue** | 1.885 | 1.746 - 2.043 | 1367289 |
| **NCI-H1975** | **TAVO412** | 1.358 | 1.086 - 1.705 | 188191 |
|  | **Amivantamab analogue** | 0.626 | 0.444 – 0.871 | 216671 |
| **NCI-H358** | **TAVO412** | 0.273 | 0.211 - 0.358 | 329916 |
|  | **Amivantamab analogue** | 0.584 | 0.550 - 0.620 | 310715 |
| **NCI-H460** | **TAVO412** | 0.267 | 0.226 - 0.317 | 239337 |
|  | **Amivantamab analogue** | 1.077 | 1.006 - 1.156 | 235874 |
| **NCI-H1299** | **TAVO412** | 0.263 | 0.179 - 0.409 | 257425 |
|  | **Amivantamab analogue** | 0.891 | 0.779 - 1.027 | 242163 |
| **NCI-H596** | **TAVO412** | 1.426 | 1.242 - 1.638 | 2366672 |
|  | **Amivantamab analogue** | 3.765 | 3.332 - 4.296 | 2529035 |

The abbreviations were: CI = confidence interval; EC_50_ = half-maximal effective concentration; nM = nanomolar; NF= no fit returned by GraphPad Prism; Abs = antibodies. The nomenclature “Binding avidity” (not “affinity”) referred to the three-arm design (2×EGFR + 1×cMET) of TAVO412 that enhances tumor cell attachment through cooperative multivalent interactions.

**Supplementary Table S3
Ligand blocking of TAVO412 in HCC827 cell line**

| **Ligand** | **EGF** | | | **HGF** | | |
| --- | --- | --- | --- | --- | --- | --- |
| **Test Abs** | **EC50 (nM)** | **95% CI (nM)** | **Span** | **EC50 (nM)** | **95% CI (nM)** | **Span** |
| **TAVO412** | 4.013 | 2.475 - 8.897 | 37373 | 0.282 | 0.254 - 0.313 | 5385 |
| **Amivantamab analogue** | 11.86 | NF | 43057 | 0.689 | 0.618 - 0.765 | 5353 |

CI = confidence interval; EC_50_ = half-maximal effective concentration; nM = nanomolar; NF= no fit returned by GraphPad Prism; Abs = antibodies

**Supplementary Table S4**

**Phosphorylation inhibition of TAVO412 in selected NSCLC cell lines**

| **Cell lines** | **Ligand** | **EGF** | | | **HGF** | | |
| --- | --- | --- | --- | --- | --- | --- | --- |
|  | **Test Abs** | **IC50**  **(nM)** | **95% CI**  **(nM)** | **Span** | **IC50 (nM)** | **95% CI**  **(nM)** | **Span** |
| **NCI-H292** | **TAVO412** | 0.941 | 0.682 - 1.237 | 91 | 0.568 | 0.209 - 1.034 | 66 |
|  | **Amivantamab analogue** | 2.796 | NF | 115 | 0.430 | NF | 88 |
| **HCC827** | **TAVO412** | 15.97 | NF | 50 | 0.862 | 0.588 – 1.210 | 94 |
|  | **Amivantamab analogue** | 16.93 | 12.18 - 40.16 | 52 | 1.157 | 0.924 - 1.385 | 67 |

The abbreviations were: CI = confidence interval; IC_50_ = half-maximal inhibition concentration; nM = nanomolar; NF= no fit returned by GraphPad Prism; Abs = antibodies

**Supplementary Table S5**

**Fc effector functions of TAVO412 in selected NSCLC cell lines**

| **Cell lines** | **Fc functions** | **ADCC** | | | **ADCP** | | | **CDC** | | | |
| --- | --- | --- | --- | --- | --- | --- | --- | --- | --- | --- | --- |
|  | **Test Abs** | **EC50 (nM)** | **95% CI (nM)** | **Span** | **EC50 (nM)** | **95% CI (nM)** | **Span** | **EC50 (nM)** | **95% CI (nM)** | **Span** |  |
| **NCI-H292** | **TAVO412** | 0.051 | 0.038 - 0.068 | 26 | 0.009 | 0.007 - 0.010 | 103 | 0.297 | 0.217 - 0.416 | 59 |  |
|  | **Amivantamab analogue** | 0.114 | NF | 24 | 0.012 | 0.010 - 0.015 | 98 | 4.011 | NF | 7 |  |
| **HCC827** | **TAVO412** | 0.066 | 0.033 - 0.368 | 11 | 0.112 | 0.087 - 0.142 | 98 | 3.139 | 2.593 – 4.177 | 52 |  |
|  | **Amivantamab analogue** | 0.483 | NF | 9 | 0.126 | 0.092 - 0.170 | 93 | 7.154 | 5.532 – 49.98 | 19 |  |
| **NCI-H1975** | **TAVO412** | 0.003 | 0.002 - 0.005 | 30 | 0.163 | 0.117 - 0.218 | 39 | 2.029 | 1.299 - 4.091 | 13 |  |
|  | **Amivantamab analogue** | 0.005 | 0.0004 - 0.010 | 45 | 0.125 | 0.075 - 0.228 | 33 | 84.90 | NF | 10 |  |

The abbreviations were: CI = confidence interval; EC_50_ = half-maximal effective concentration; nM = nanomolar; NF= no fit returned by GraphPad Prism; Abs = antibodies

**Supplementary Table S6**

**EGFR-TKI affects the cell binding of TAVO412 in NSCLC cell lines**

| **Cell lines** | **Treatment** | **37℃, 1h** | | | **37℃, 24h** | | |
| --- | --- | --- | --- | --- | --- | --- | --- |
|  | **Test Abs** | **EC50 (nM)** | **95% CI (nM)** | **Span** | **EC50 (nM)** | **95% CI (nM)** | **Span** |
| **NCI-H1975** | **TAVO412** | 0.283 | 0.241 - 0.330 | 184197 | 0.574 | 0.539 - 0.611 | 40486 |
|  | **TAVO412+**  **Osimertinib (1 nM)** | 0.350 | 0.293 - 0.417 | 235915 | 0.648 | 0.616 - 0.682 | 43485 |
|  | **TAVO412+**  **Osimertinib (20 nM)** | 0.371 | 0.307 - 0.447 | 232073 | 0.653 | 0.583 - 0.732 | 67284 |
|  | **TAVO412+**  **Osimertinib (125 nM)** | 0.435 | 0.337 - 0.558 | 225056 | 0.732 | 0.655 - 0.825 | 105836 |
| **HCC827** | **TAVO412** | 1.389 | 1.211 - 1.597 | 1686603 | 9.985 | NF | 690356 |
|  | **TAVO412+**  **Lazertinib (0.3 nM)** | 1.598 | 1.434 - 1.777 | 1594561 | 7.097 | NF | 484898 |
|  | **TAVO412+**  **Lazertinib (3 nM)** | 0.758 | NF | 471862 | 4.306 | 3.802 - 4.876 | 413188 |
|  | **TAVO412+**  **Lazertinib (100 nM)** | 0.580 | 0.517 - 0.645 | 479532 | 1.718 | 1.530 - 1.919 | 261843 |

The abbreviations were: CI = confidence interval; EC_50_ = half - maximal effective concentration; nM = nanomolar; NF= no fit returned by GraphPad Prism; Abs = antibodies; TKI, tyrosine kinase inhibitors

**Supplementary Table S7**

**EGFR-TKI affects the ADCC effector function of TAVO412 in NSCLC cell lines**

| **Cell lines** | **Fc function** | **ADCC** | | |
| --- | --- | --- | --- | --- |
|  | **Test Abs** | **EC50 (nM)** | **95% CI (nM)** | **Span** |
| **NCI-H1975** | TAVO412 | 0.004 | NF | 28 |
|  | TAVO412 + Osimertinib (20 nM) | 0.010 | 0.006 - 0.014 | 32 |
|  | TAVO412 + Osimertinib (125 nM) | 0.009 | 0.005 - 0.013 | 29 |
| **HCC827** | TAVO412 | 0.057 | NF | 11 |
|  | TAVO412 + Lazertinib (3 nM) | 0.068 | 0.019 - 0.697 | 10 |
|  | TAVO412 + Lazertinib (100 nM) | 0.040 | 0.024 - 0.063 | 17 |

The abbreviations were: CI = confidence interval; EC_50_ = half-maximal effective concentration; nM = nanomolar; NF= no fit returned by GraphPad Prism; Abs = antibodies; TKI, tyrosine kinase inhibitors
